# The drebrin/EB3 pathway drives invasive activity in prostate cancer

**DOI:** 10.1038/onc.2017.45

**Published:** 2017-03-20

**Authors:** A E Dart, D C Worth, G Muir, A Chandra, J D Morris, C McKee, C Verrill, R J Bryant, P R Gordon-Weeks

**Affiliations:** 1 The MRC Centre for Developmental Neurobiology, King's College London, New Hunts House, Guy's Campus, London, UK; 2 Urology, King's College Hospital, London, UK; 3 Cellular Pathology, 2nd floor North Wing, St. Thomas' Hospital, London, UK; 4 Division of Cancer Studies, New Hunt’s House, Guy’s Campus, King’s College London, London, UK; 5 Oxford Institute for Radiation Oncology, Churchill Hospital, University of Oxford, Oxford, UK; 6 Nuffield Department of Surgical Sciences, University of Oxford, Oxford, UK

## Abstract

Prostate cancer is the most common cancer in men and the metastatic form of the disease is incurable. We show here that the drebrin/EB3 pathway, which co-ordinates dynamic microtubule/actin filament interactions underlying cell shape changes in response to guidance cues, plays a role in prostate cancer cell invasion. Drebrin expression is restricted to basal epithelial cells in benign human prostate but is upregulated in luminal epithelial cells in foci of prostatic malignancy. Drebrin is also upregulated in human prostate cancer cell lines and co-localizes with actin filaments and dynamic microtubules in filopodia of pseudopods of invading cells under a chemotactic gradient of the chemokine CXCL12. Disruption of the drebrin/EB3 pathway using BTP2, a small molecule inhibitor of drebrin binding to actin filaments, reduced the invasion of prostate cancer cell lines in 3D *in vitro* assays. Furthermore, gain- or loss-of-function of drebrin or EB3 by over-expression or siRNA-mediated knockdown increases or decreases invasion of prostate cancer cell lines in 3D *in vitro* assays, respectively. Finally, expression of a dominant-negative construct that competes with EB3 binding to drebrin, also inhibited invasion of prostate cancer cell lines in 3D *in vitro* assays. Our findings show that co-ordination of dynamic microtubules and actin filaments by the drebrin/EB3 pathway drives prostate cancer cell invasion and is therefore implicated in disease progression.

## Introduction

Drebrin is a filamentous actin (F-actin)-binding protein with roles in neuronal development and synaptic plasticity.^1^ Drebrin couples dynamic microtubules to F-actin in filopodia during neuritogenesis and in dendritic spines by binding to the microtubule-binding +TIP protein EB3.^2, 3^ There are two domains in the N-terminal half of drebrin, which independently bind to F-actin.^4^ These two domains act co-operatively to bundle F-actin but this activity is repressed by an intramolecular interaction that is relieved by Cdk5 phosphorylation of S142.^4^ Drebrin has a role in oculomotor neuron migration,^5^ and phospho-mimetic and phospho-dead mutants of S142 enhance and inhibit neuritogenesis, respectively.^4^ Furthermore, either mutant inhibits cerebral cortical neuronal migration^6^ and migration of olfactory bulb precursor neurons,^7^ implying that regulation of this phosphorylation is crucial to neuronal migration. Cell migration is important for cancer progression and the demonstrated role for drebrin in neuronal migration therefore prompted us to investigate a possible role for the drebrin/EB3 pathway in cancer cell invasion.

Prostate cancer is the most common malignancy diagnosed in men in the Western world and the second leading cause of male cancer-related death.^8^ Malignant cells most likely arise from either a failure of the appropriate differentiation of basal epithelial cells that normally give rise to both basal and luminal epithelial cells, or from a failure of luminal cell differentiation,^9, 10, 11^ and processes such as epithelial-to-mesenchymal transition result in the acquisition of an invasive cancer cell phenotype.^12^ Prostate cancer cells commonly metastasise to bone and there is evidence that the chemokine CXCL12, acting through its cognate receptor CXCR4, plays a role in bone metastasis.^13, 14, 15, 16^ Here we show that drebrin, an actin filament-binding protein that also binds to the CXCR4 receptor,^17^ and EB3 a microtubule +TIP protein in the drebrin/EB3 pathway, contribute to prostate cancer cell invasion.

## Results

### Drebrin and pS142-drebrin are upregulated in malignant prostate

**Figure 1:**
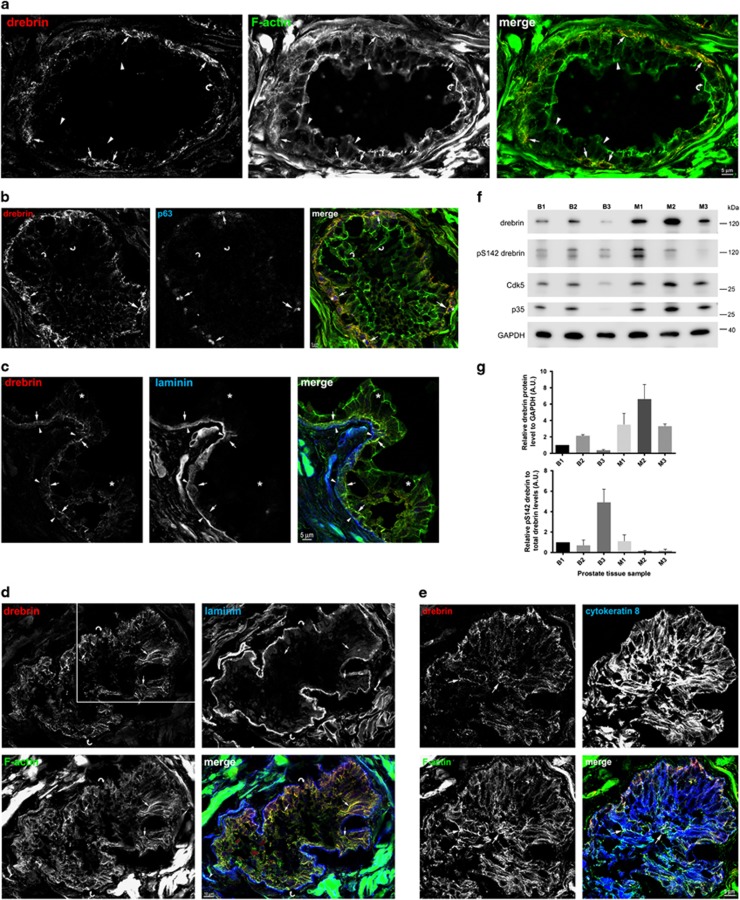
Drebrin is expressed in basal epithelial cells in non-malignant human prostate and upregulated in luminal epithelial cells in human prostate cancer tissue. (**a**) Drebrin is expressed by a population of cells in the glandular epithelium of benign human prostate hyperplasia, where it co-localizes with F-actin. Immunofluorescence images of human prostate tissue labelled with an antibody to drebrin and phalloidin to label F-actin. Drebrin in basal cells co-localizes with F-actin (arrows). Luminal cells (arrowheads) do not contain drebrin and therefore drebrin is not associated with the F-actin in the terminal junctional web of luminal cells (curved arrow). (**b**) Drebrin-expressing epithelial cells also express the transcription factor protein p63, a basal cell marker, in their nucleus. Immunofluorescence images of human non-malignant prostate tissue labelled with antibodies to drebrin and p63 and with phalloidin to label F-actin. Drebrin (arrows) is expressed in cells that also express nuclear p63 (asterisks) and co-localizes with F-actin (arrows). Drebrin is not associated with the F-actin in the terminal junctional web of luminal cells (curved arrows). (**c**) Drebrin is expressed by cells that contact the basal lamina in the glandular epithelium of non-malignant human prostate. Immunofluorescence images of human prostate tissue labelled with antibodies to drebrin and to laminin to label the basal lamina, and with phalloidin to label F-actin. Drebrin is present in basal cells (arrows) that contact the basal lamina (arrowheads) and co-localizes with F-actin. Luminal epithelial cells do not express drebrin (asterisks). (**d**) Drebrin is upregulated in luminal epithelial cells in the glands of malignant human prostate tissue. Immunofluorescence images of malignant human prostate tissue labelled with antibodies to drebrin and laminin, to label the basal lamina and phalloidin to label F-actin. In luminal epithelial cells drebrin is particularly localized to baso-lateral bundles of F-actin (arrows). The glands have a disorganized architecture and there are no F-actin terminal webs visible. Notably the basal lamina appears to be deficient as indicated (curved arrows). (**e**) Luminal epithelial cells upregulating drebrin express cytokeratin 8, confirming their luminal phenotype. Immunofluorescence images of malignant human prostate tissue labelled with antibodies to drebrin and cytokeratin 8, and with phalloidin to label F-actin. Drebrin is expressed in cells throughout the acinus including luminal cells that also express cytokeratin 8, a marker for luminal cells (arrows). Drebrin in these cells co-localizes with F-actin. The acinus is the same one shown in **d** (white box area) but from an adjacent section. (**f**) Immunoblot of three individual cases of benign human prostate hyperplasia (B1–B3) and three individual cases of human prostate cancer (M1–M3) probed with antibodies to drebrin, pS142-drebrin, Cdk5, p35 and GAPDH, as a loading control. Drebrin is expressed in benign prostate tissue and upregulated in malignant prostate tissue. Despite upregulation of Cdk5 and p35 in malignant prostate, pS142-drebrin is variably expressed in malignant prostate. The lower band of the pS142-drebrin blot is a breakdown product of drebrin. (**g**) Quantification of relative drebrin and pS142-drebrin protein levels normalized to GAPDH and drebrin, respectively, from immunoblots of benign (B1–B3) and malignant (M1–M3) prostate tissue. Error bars are mean±s.e.m. from three replicate samples.

In sections of benign human prostate, drebrin co-localizes with F-actin in a population of epithelial cells (Figure 1a). These cells express the basal cell marker p63, and are therefore likely to be basal prostate epithelial cells (Figure 1b).^11, 18^ Consistent with this identity, drebrin-expressing cells contact the basal lamina that surrounds the glands, as revealed by labelling with laminin antibodies (Figure 1c). Luminal cells in the glands do not express drebrin but, unlike the basal cells, contain bundles of vimentin intermediate filaments and cytokeratin 8 (not shown).

In sections of human prostate cancer tissue, in addition to drebrin-positive basal epithelial cells in areas with benign glands, we found glands in which luminal epithelial cells also expressed drebrin (Figure 1d). Luminal epithelial cells expressing drebrin also expressed the luminal cell markers cytokeratin 8 (Figure 1e) and vimentin (not shown), thereby confirming their identity.^11^ We confirmed the presence of drebrin in malignant and benign prostate by immunoblotting and found that the levels of drebrin were significantly greater in malignant tissue (Figures 1f and g). We also detected pS142-drebrin in malignant samples, although, despite correspondingly increased levels of p35 and Cdk5 in malignant tissue, these were not always higher than in benign prostate (Figures 1f and g).

To investigate the expression of drebrin and pS142-drebrin in a larger cohort of benign and malignant prostate tissue samples we analysed a tissue microarray of human prostate sections from patients who had undergone radical prostatectomy as primary treatment for prostate cancer. We immunolabelled the tissue microarray with either drebrin or pS142-drebrin antibodies and observed that drebrin and pS142-drebrin localized to the cytoplasm and plasma membrane of both benign and malignant prostate epithelial cells (Supplementary Figure S1A–D). Cytoplasmic drebrin expression and membranous pS142-drebrin expression were significantly higher in prostate cancer samples compared with benign samples (Supplementary Figure S1E and F). To uncover potential associations between altered drebrin expression and clinical outcome in prostate cancer we interrogated data (mRNA expression changes and copy number variations) from the publically available MSKCC Prostate Cancer Genomics Data Portal data set.^19^ We found that both increased mRNA expression and copy number gain of drebrin, Cdk5 and Cdk5R1 (p35, the regulatory subunit of Cdk5), occur significantly more frequently in prostate cancer metastasis samples (*n*=37) than in primary (that is, organ-confined, *n*=179) prostate cancer samples (Supplementary Figure S2). In addition, patients in this cohort with prostate cancer containing a genetic alteration in Cdk5R1 (p35) had a significantly reduced disease-free survival following treatment (Supplementary Figure S3), and a similar trend was observed for drebrin and Cdk5 although these latter two proteins did not reach statistical significance. Significant positive correlations were observed between mRNA expression levels of drebrin, Cdk5 and Cdk5R1 (Supplementary Figure S3). Taken together these results suggest that prostate cancer metastases more frequently harbour genetic changes associated with over-expression of drebrin, and proteins regulating drebrin’s actin bundling activity, as compared with primary prostate cancer samples, raising the possibility that these events might promote (or at least be associated with) prostate cancer progression to a metastatic phenotype.

### Drebrin and pS142-drebrin are upregulated in prostate cancer cell lines and co-localize with dynamic microtubules and F-actin in filopodia

**Figure 2:**
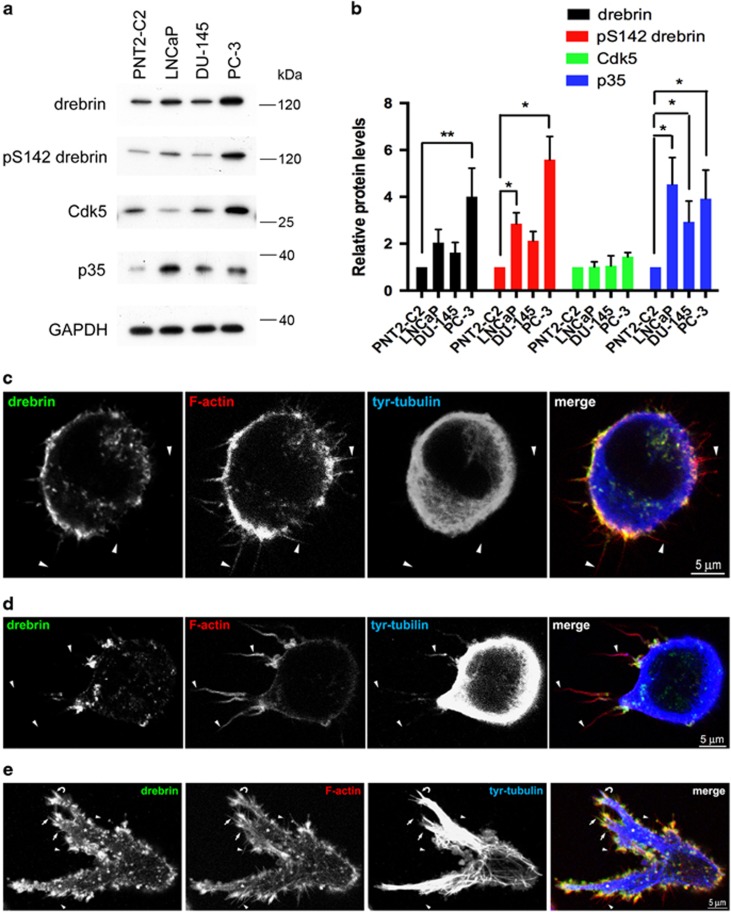
Drebrin and pS142-drebrin are upregulated in human prostate cancer cell lines. (**a**) Immunoblots of human prostate epithelial cell lines probed with antibodies against drebrin, pS142-drebrin, Cdk5, p35 and, as a loading control, GAPDH. Drebrin is expressed at moderate levels in the normal human prostate epithelial cell line PNT2-C2 and the human prostate cancer cell line DU-145, and at higher levels in the human prostate cancer cell lines LNCaP and PC-3. There is a tight correlation between the levels of drebrin and pS142-drebrin. p35 is highly upregulated in prostate cancer cell lines compared to PNT2-C2 cells. (**b**) Quantification of relative protein levels from immunoblots of PNT2-C2, LNCaP, DU-145 and PC-3 cell lysates probed with antibodies against drebrin, pS142-drebrin, Cdk5 and p35. Error bars are mean±s.e.m. from at least three independent experiments. Significant differences: *P<0.05; **P<0.01. (**c**–**e**) Immunofluorescence images of PC-3 cells in 3D Matrigel in a CXCL12 gradient in chemotaxis chambers. The source of CXCL12 is to the left. Cells were labelled with antibodies against drebrin and tyrosinated α-tubulin (tyr-tubulin), to identify dynamic microtubules, and with phalloidin, to label F-actin. (**c**) An unpolarized, rounded cell with F-actin containing filopodia extended in all directions (arrowheads). Drebrin is distributed in the cell cortex co-localizing with F-actin. (**d**) A polarized cell before pseudopod production. The longest filopodia are extended on the side of the cell facing the CXCL12 gradient (arrowheads). Dynamic microtubules (tyr-tubulin) have entered the filopodia facing the gradient and drebrin has re-located to their base. (**e**) A polarized cell with several pseudopods (asterisks) covered in filopodia (arrowheads). Drebrin strongly co-localizes with F-actin, particularly at the base of filopodia (arrowheads). Large bundles of dynamic microtubules (tyr-tubulin) enter pseudopods and single or small bundles of microtubules enter some filopodia (curved arrow).

We screened a panel of human prostate cancer cell lines (LNCaP, DU-145, PC-3) and a non-malignant human prostate epithelial cell line (PNT2-C2) for drebrin expression (Figure 2a). We detected moderate levels of drebrin in PNT2-C2 cells and higher levels in DU-145 cells and LNCaP cells. PC-3 cells had the highest levels of drebrin compared to PNT2-C2 cells (Figures 2a and b). To investigate the levels of Cdk5-phosphorylated drebrin we used pS142-drebrin antibodies.^4^ This showed that pS142-drebrin is present in all prostate cancer cell lines examined and that the levels are proportional to the levels of drebrin (Figures 2a and b). The levels of pS142-drebrin do not correlate with Cdk5R1 (p35), the regulatory subunit of Cdk5 or with Cdk5 (Figure 2b).

We cultured PC-3 cells in 3D Matrigel in a gradient of CXCL12, since they express CXCR4, the cognate receptor for this chemokine.^14^ PC-3 cells were initially rounded and extended filopodia in all directions (Figure 2c). In these cells, drebrin was distributed around the cell cortex co-localizing with F-actin (Figure 2c). Subsequently, filopodia on the side of the cell facing the gradient became noticeably longer than those on the opposite side (Figure 2d). Drebrin and dynamic microtubules, identified by immunolabelling with an antibody to dynamic microtubules,^20^ became localized to these longer filopodia (Figure 2d). Eventually, most cells became elongated and polarized, with large, filopodia-covered pseudopods containing drebrin and dynamic microtubules (Figure 2e).

To explore whether the CXCL12/CXCR4 axis signals directly to the drebrin/EB3 pathway we looked for an effect of CXCL12 on drebrin phosphorylation at S142. We treated PC-3 cells in culture with CXCL12 and confirmed previous reports^21^ that CXCL12 induces extracellular signal-regulated kinase (ERK) phosphorylation (Supplementary Figure S4). In addition, we found a slight, time-dependent decline in pS142-drebrin levels, although this did not reach statistical significance (Supplementary Figure S4).

### BTP2, an inhibitor of drebrin binding to F-actin, reduces PC-3 cell invasion

**Figure 3:**
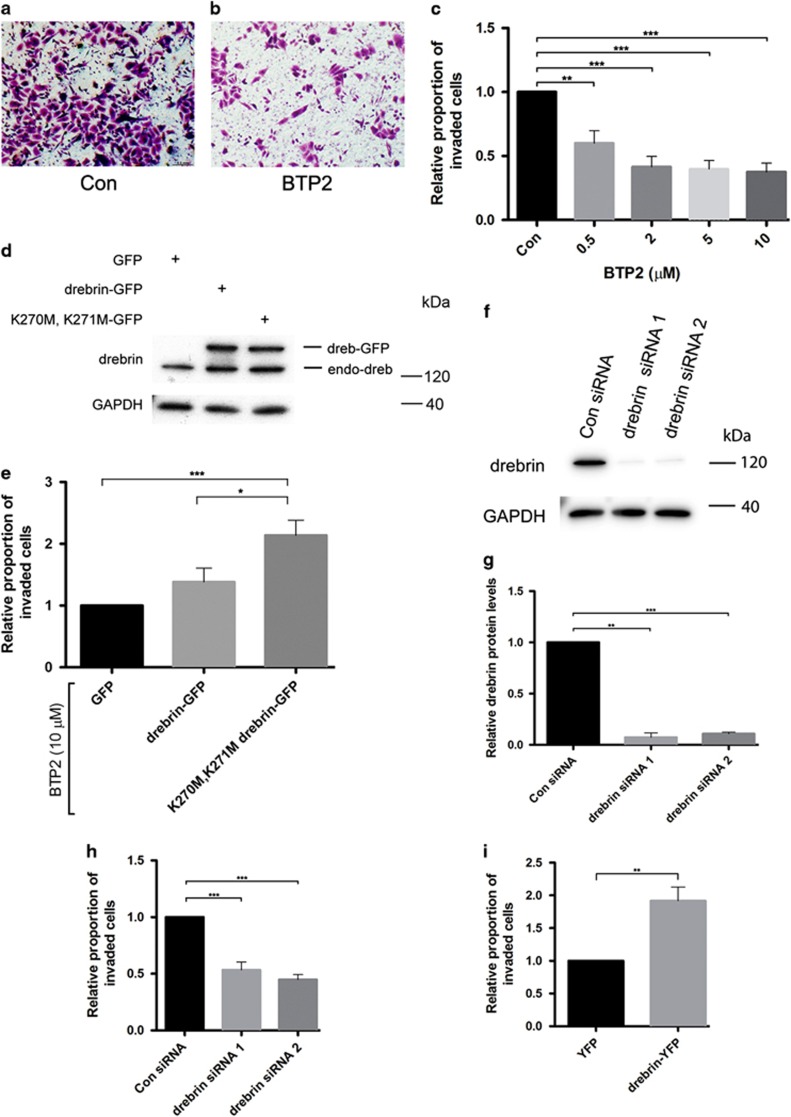
BTP2 and drebrin knockdown inhibit PC-3 cell invasion in 3D assays in the presence of CXCL12 chemotactic gradients. (**a**, **b**) Micrographs of cresyl violet-stained PC-3 cells on the lower surface of the insert membrane from 3D invasion assays using control (Con) or BTP2 (5 μM) treated cultures. (**c**) Effects of BTP2 on PC-3 cell invasion in a 3D invasion assay. PC-3 cells were incubated in suspension with vehicle (Con) or with different concentrations of BTP2 for 1 h before re-addition of vehicle or BTP2 and then plating onto Matrigel in the Transwell insert. After 48 h, cells on the lower surface of the insert membrane were stained with cresyl violet and counted. Error bars are mean±s.e.m. from four independent experiments each of two replicates per condition. Significant differences: **P<0.01; ***P<0.001. (**d**) PC-3 cells transfected with GFP, wild-type drebrin-GFP or K270M, K271M drebrin-GFP show similar levels of protein expression. Immunoblots of transfected PC-3 cells were probed with antibodies against drebrin and GAPDH. The drebrin antibody recognizes endogenous drebrin (endo-dreb) and the expressed protein (dreb-GFP). (**e**) Effects of a BTP2-drebrin-binding mutant on BTP2 inhibition of PC-3 cell invasion in a 3D invasion assay. Cells were transfected with GFP, wild-type drebrin-GFP or K270M, K271M drebrin-GFP and incubated with BTP2 (10 μM) for 1 h followed by re-addition of BTP2 (10 μM) before seeding onto Matrigel in the Transwell insert. After 48 h, cells on the lower surface of the insert membrane were stained with cresyl violet and counted. Error bars are mean±s.e.m. from four independent experiments each of two replicates per condition. Significant differences: *P<0.05; ***P<0.001. (**f**) Immunoblots of PC-3 cells transfected with either control siRNA (Con siRNA) or drebrin siRNA 1 or drebrin siRNA 2 and probed with antibodies against drebrin and GAPDH, as a loading control. (**g**) Quantification of relative drebrin protein levels from immunoblots of PC-3 cells transfected with control siRNA (Con siRNA) or drebrin siRNA 1 or drebrin siRNA 2. Drebrin-specific siRNAs knockdown drebrin levels by >85% compared to control. Error bars are mean±s.e.m. from two independent experiments. Significant differences: **P<0.01, ***P<0.001. (**h**) Effects of siRNA knockdown of drebrin on PC-3 cell invasion in a 3D invasion assay. Cells were transfected with control siRNA (Con siRNA) or drebrin siRNA 1 or drebrin siRNA 2 before seeding onto Matrigel in the Transwell insert. After 48 h, cells on the lower surface of the insert membrane were stained with cresyl violet and counted. Error bars are mean±s.e.m. from four independent experiments each of two replicates per condition. Significant difference: ***P<0.001. (**i**) Effects of over-expression of drebrin on PC-3 cell invasion in a 3D invasion assay. Cells were transfected with drebrin-YFP or YFP alone before seeding onto Matrigel in the Transwell insert. After 48 h, cells on the lower surface of the insert membrane were stained with cresyl violet and counted. Error bars are mean±s.e.m. from three independent experiments each of two replicates per condition. Significant difference: **P<0.01.

To assess whether drebrin has a role in PC-3 cell invasion we tested the effect of BTP2, a small molecule inhibitor of drebrin binding to F-actin,^22^ in a 3D Matrigel Transwell invasion assay using CXCL12 as a chemoattractant. BTP2 produced a dose-dependent reduction in the number of PC-3 cells that passed through the insert filter compared to vehicle-treated cells (Figures 3a–c). The lowest concentration of BTP2 used (0.5 μM) produced inhibition and maximal inhibition of 63% occurred with 10 μM BTP2 (Figure 3c). We checked whether changes in cell viability or adhesion to Matrigel might contribute to inhibition of invasion produced by BTP2 (Supplementary Figure S5A and B). This showed that BTP2 reduces cell viability and cell adhesion only at 10 μM and therefore could not account for the inhibition of PC-3 cell invasion.

Binding of BTP2 to drebrin requires two adjacent lysines: K270 and K271 in human drebrin.^22^ Mutation of these lysines to methionine abrogates BTP2 binding but does not affect drebrin binding to F-actin.^22^ We used the K270M, K271M drebrin mutant to directly test the role of drebrin in the inhibition of invasion produced by BTP2. We transfected PC-3 cells with wild-type drebrin-green fluorescent protein (GFP), K270M, K271M drebrin-GFP or GFP and compared the effects of BTP2 treatment on invasion of these cells in the 3D invasion assay. Immunoblotting showed similar levels of expression with the three constructs (Figure 3d). The K270M, K271M drebrin-GFP construct rescued the BTP2 inhibitory effect on PC-3 cell invasion in 3D assays (Figure 3e), showing that the action of BTP2 on PC-3 invasion is mediated by binding to drebrin. Expression of the K270M, K271M drebrin-GFP construct produced the same relative proportion of invaded cells as expression of drebrin-GFP and both constructs became phosphorylated on S142 (Supplementary Figure S6A and B) suggesting that K270M, K271M drebrin-GFP behaves like wild-type drebrin.

### Drebrin knockdown or over-expression inhibits or enhances prostate cancer cell invasion respectively

Transfection of PC-3 or LNCaP C4-2B cells with either of two, independent, drebrin-specific siRNAs reduced drebrin levels in PC-3 cells by >85%, as demonstrated by quantitative immunoblotting (Figures 3f and g; Supplementary Figure S7A and B). Drebrin knockdown inhibited PC-3 and LNCaP C4-2B cell invasion in 3D assays to a similar extent as that seen with BTP2 (Figure 3h; Supplementary Figure S7C). Cell viability assays showed that the effect of drebrin-specific siRNA is not due to cell death (Supplementary Figure S7C). Conversely, expression of drebrin-YFP in PC-3 cells increased cell invasion in 3D assays compared to expression of YFP alone (Figure 3i). These findings independently confirm a role for drebrin in PC-3 cell invasion.

### BTP2 treatment or drebrin knockdown inhibits PC-3 cell invasion by attenuating polarization and the chemotactic response to a CXCL12 gradient

**Figure 4:**
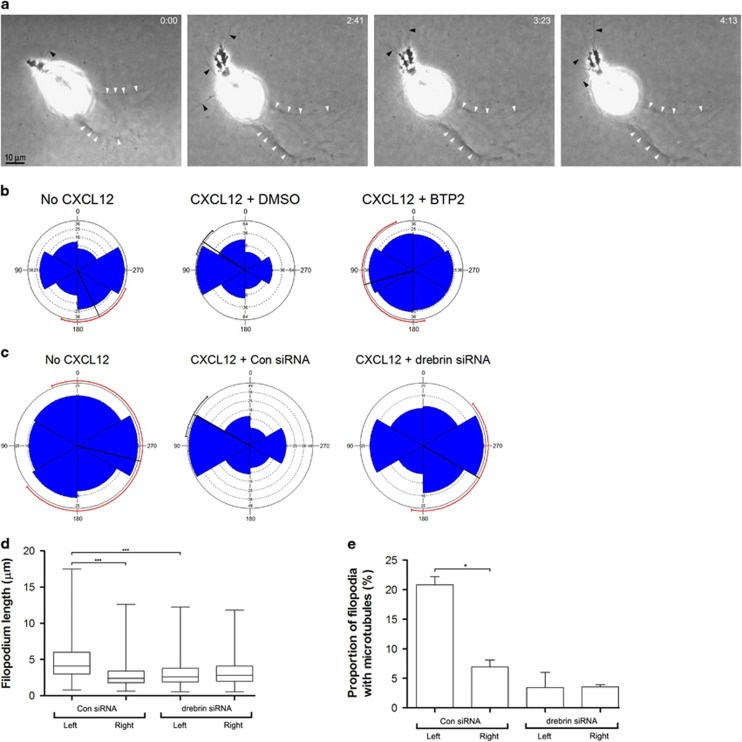
Drebrin knockdown inhibits PC-3 cell invasion by attenuating polarization and the chemotactic response to a CXCL12 gradient. (**a**) Selected frames from phase contrast videos of PC-3 cells in Matrigel in chemotaxis chambers. The source of CXCL2 is to the left. After 48 h, PC-3 cells have become polarized, extending pseudopods with filopodia (arrowheads) and have oriented towards the CXCL12 gradient. The cell has moved across the field from right to left, as indicated by a trail of degraded Matrigel (white arrowheads). Elapsed time in hours and minutes is indicated in the top right-hand corner. (b, c) Polar plots showing the invasive response of PC-3 cells to a gradient of CXCL12 in 3D Matrigel culture. Cells were treated with: (**b**) vehicle (DMSO) or 5 μM BTP2, or (**c**) drebrin siRNA or control siRNA (Con siRNA) and cultured in Matrigel in chemotaxis chambers with or without a CXCL12 gradient. The source of CXCL12 is to the left. The orientation of degradation trails with respect to the gradient of CXCL12 was recorded after 48 h in culture. At least 20 cells were counted from each condition in each of three independent experiments. The radial black line shows the mean for the data and the circumferential line the 95% tolerance limits. A red circumferential line indicates that the confidence limits are unreliable. (**d**) Filopodia length of rounded PC-3 cells transfected with control siRNA (Con siRNA) or drebrin siRNA in 3D Matrigel in a concentration gradient of CXCL12 at 24 h. Filopodia lengths on the gradient-facing, left side of the cell were compared with those on the opposite, right side of the cell. Numbers are for three independent experiments. The filopodia of at least five cells were counted from each condition in each experiment. Significant difference: ***P<0.001. (**e**) Proportion of filopodia containing dynamic microtubules in rounded PC-3 cells transfected with control siRNA (Con siRNA) or drebrin siRNA in 3D Matrigel in a concentration gradient of CXCL12 at 24 h. Filopodia on the gradient-facing, left side of the cell were compared with those on the opposite, right side of the cell. Numbers are mean±s.e.m. for two independent experiments. The filopodia of at least 10 cells were counted from each condition in each experiment. Significant difference: *P<0.05.

We used phase contrast microscopy to image PC-3 cells in chemotaxis chambers with CXCL12 gradients (Figure 4). After 48 h in the chemotactic gradient we found examples of cells migrating in the direction of the CXCL12 gradient while leaving behind a degradation trail in the Matrigel that was visible in phase contrast (Figure 4a, white arrowheads).^23^ In the absence of a CXCL12 gradient the orientation of degradation trails of untreated PC-3 cells or cells transfected with control siRNA showed a slight preference in one direction but the confidence limits were large and unreliable (Figures 4b and c). In the presence of a CXCL12 gradient, degradation trails of vehicle-treated PC-3 cells or cells transfected with control siRNA were preferentially oriented toward the gradient (Figures 4b and c) whereas the degradation trails of PC-3 cells treated with BTP2 or transfected with drebrin siRNA showed no preferred orientation (Figures 4b and c).

To investigate whether drebrin affects polarization of PC-3 cells in 3D in the presence of CXCL12 gradients we compared the length of filopodia on the gradient-facing side of rounded cells, before they had produced pseudopods, with the length of filopodia on the opposite side (Figure 4d). After 24 h in the chemotactic gradient, PC-3 cells transfected with control siRNA had longer filopodia on the gradient-facing side of the cell than on the opposite side (Figure 4d). In contrast, PC-3 cells transfected with drebrin siRNA, showed no difference in filopodia length between the two sides of the cell (Figure 4d). This finding suggests that drebrin contributes to filopodia polarization during the early stages of cell polarization. Consistent with this idea, drebrin becomes localized particularly to the longer filopodia on the side of the cell facing the CXCL12 gradient (Figure 2d) and these filopodia are more commonly penetrated by dynamic microtubules (Figures 2d and 4e).

Together these findings suggest that drebrin plays a role in the chemotactic response to CXCL12. To determine whether drebrin also plays a role in prostate cancer cell motility, PC-3 cells were plated into 6-well dishes coated with fibronectin and imaged by phase contrast microscopy in the presence of DMSO (vehicle control) or different concentrations of BTP2 to inhibit drebrin binding to F-actin. Cell motility was measured from 100 images captured every 10 min. This showed that BTP2 maximally reduces PC-3 cell motility by about 50% (Supplementary Figure S8), implying that drebrin also contributes to cell motility.

### EB3M, a dominant-negative construct of the drebrin/EB3 pathway, inhibits PC-3 cell invasion

**Figure 5:**
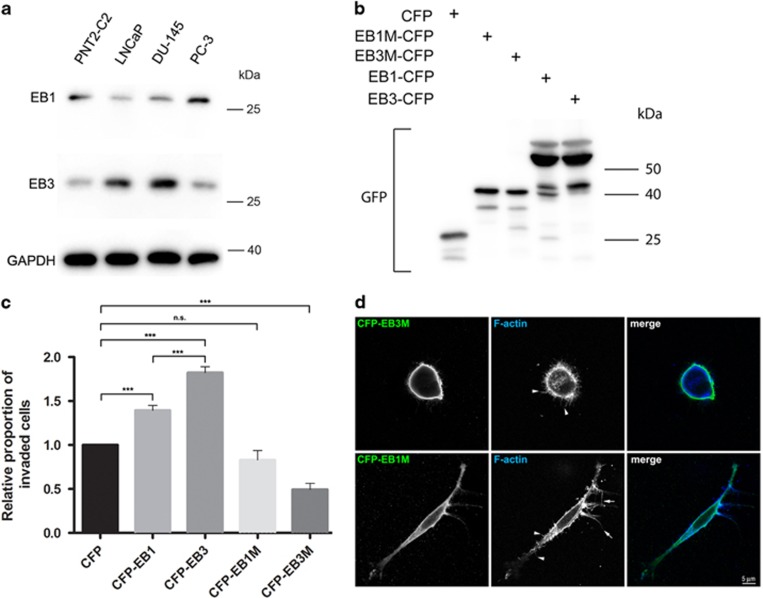
Evidence for a role for drebrin/EB3 interactions in PC-3 cell invasion. (**a**) Immunoblots of a normal human prostate epithelial cell line (PNT2-C2) and human prostate cancer cell lines probed with antibodies against EB1, EB3 and, as a loading control, GAPDH. (**b**) Immunoblots of PC-3 cells expressing CFP, CFP-EB1M, CFP-EB3M, CFP-EB1 or CFP-EB3 probed with an antibody to GFP that cross-reacts with CFP. Expression levels of related constructs are similar. (**c**) Effects of transfection of PC-3 cells with CFP, CFP-EB1 CFP-EB3, CFP-EB1M or CFP-EB3M on cell invasion in a 3D invasion assay in the presence of CXCL12 chemotactic gradients. After 48 h, cells on the lower surface of the insert membrane were labelled with an antibody to GFP and counted using confocal fluorescence microscopy. Error bars are mean±s.e.m. from four independent experiments each of two replicates per condition. Significant difference: ***P<0.001. NS, not significant. (**d**) Immunofluorescence micrographs of PC-3 cells labelled with a GFP antibody that cross-reacts with CFP, and with phalloidin to label F-actin. Cells were transfected with CFP-EB1M or CFP-EB3M, embedded in Matrigel and exposed to a CXCL12 gradient. PC-3 cells expressing CFP-EB3M are rounded and produce short filopodia (arrowheads) but no pseudopods. In contrast, PC-3 cells expressing CFP-EB1M become polarized and extend pseudopods bearing long, tapered filopodia (arrows) and short, uniform filopodia (arrowheads).

Drebrin binds to the microtubule +TIP protein EB3, but not to the closely related family member EB1, and this interaction couples dynamic microtubules to F-actin.^2, 4, 24, 25^ The drebrin-binding site is in the mid-region of EB3 and a construct encoding this region (EB3M) acts as a dominant-negative.^2^ The relative levels of EB1 and EB3 vary between prostate cancer cell lines (Figure 5a). Lower levels of EB3 than EB1 were found in PNT2-C2 and PC-3 cells, but higher levels of EB3 than EB1 were observed in LNCaP and DU-145 cells (Figure 5a). We tested the effects of cyan fluorescent protein (CFP), CFP-EB1, CFP-EB1M (encoding the homologous mid-region of EB1), CFP-EB3 and CFP-EB3M on PC-3 cell invasion through Matrigel (Figures 5b and c). We obtained similar levels of protein expression of related constructs (Figure 5b). Expression of CFP-EB1 or CFP-EB3 increased PC-3 cell invasion through Matrigel in 3D assays by 39 and 80%, respectively, compared to CFP alone. In contrast, CFP-EB3M reduced PC-3 cell invasion by 51%, whereas CFP-EB1M reduced PC-3 cell invasion by only 17%, which was not significant (Figure 5c). Transfection of PC-3 cells with CFP-EB3M, but not with CFP-EB1M, dramatically altered their morphology (Figure 5d). CFP-EB3M-expressing cells were distinctly rounded with no pseudopods, which might contribute to their invasion defect (Figure 5d). These results suggest that an interaction between drebrin and EB3 is important for PC-3 cell invasion of Matrigel in CXCL12 gradients.

### EB1 and EB3 contribute to prostate cancer cell invasion in response to a CXCL12 gradient

**Figure 6:**
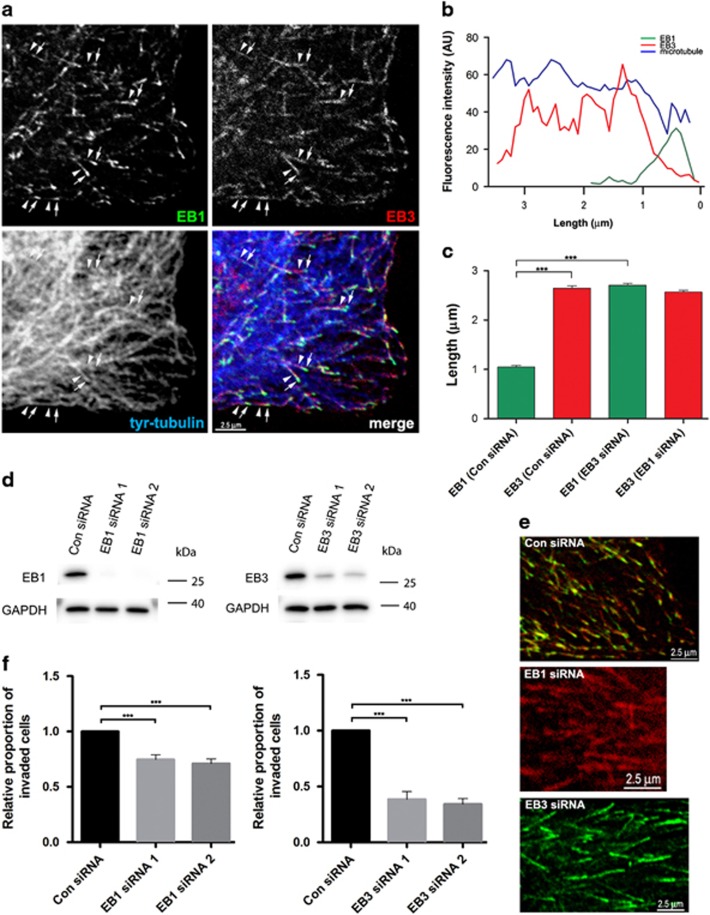
EB1 and EB3 contribute to PC-3 cell invasion. (**a**) Immunofluorescence images of a human prostate cancer PC-3 cell grown on a glass coverslip and labelled with antibodies against EB1, EB3 and tyrosinated α-tubulin (tyr-tubulin), to identify dynamic microtubules. EB1 localizes to the tip of dynamic microtubules (arrows) and has a shorter and more distal localization than EB3 (arrowheads). (**b**) Fluorescence intensity line plots of EB1 (green), EB3 (red) and tyr-tubulin (blue) in PC-3 cells. (**c**) Quantitative analysis of the length distribution of EB1 and EB3 on dynamic microtubules measured from fluorescent intensity line plots in PC-3 cells transfected with either control siRNA (Con siRNA) or EB1 siRNA 1 and 2 or EB3 siRNA 1 and 2 and probed with antibodies against EB1 and EB3. Error bars are mean±s.e.m. from three independent experiments each of at least thirty measurements. Significant difference: ***P<0.001. (**d**) Immunoblots of PC-3 cells transfected with control siRNA (Con siRNA) or EB1 siRNA 1 or 2 or EB3 siRNA 1 or 2 and probed with antibodies against EB1, EB3 and GAPDH. EB1 siRNA specifically knocks down EB1 levels by >95% compared to control and EB3 siRNA specifically knocks down EB3 levels by >55% compared to control. (**e**) Immunofluorescence images of human prostate cancer PC-3 cells grown on glass coverslips and labelled with antibodies against EB1 and EB3. Cells were transfected with control siRNA or with EB1 siRNA 1 and 2 or EB3 siRNA 1 and 2. In control cells (Con siRNA), EB1 localizes to the tip of dynamic microtubules and has a shorter and more distal localization than EB3. In cells lacking EB1 (EB1 siRNA), the distribution of EB3 along microtubules appears normal whereas in cells lacking EB3 (EB3 siRNA), the distribution of EB1 along the microtubule increases. (**f**) Effects of EB1 and EB3 knockdown with siRNA on PC-3 cell invasion in a 3D invasion assay in the presence of CXCL12 chemotactic gradients. Cells were transfected with control siRNA (Con siRNA) or EB1 siRNA 1 or 2 or EB3 siRNA 1 or 2 before seeding onto Matrigel in the Transwell insert. After 48 h, cells on the lower surface of the insert membrane were stained with cresyl violet and counted. EB3 knockdown has a greater effect on PC-3 invasion than EB1. Error bars are mean±s.e.m. from four independent experiments each of two replicates per condition. Significant differences: **P<0.01, ***P<0.001.

The +TIP proteins EB1 and EB3 have distinct distributions at the plus-end of dynamic microtubules in PC-3 (Figure 6a) and LNCaP C4-2B cells (not shown). EB1 is located closer to the microtubule plus-end than EB3 and occupies a shorter length of the microtubule (Figures 6a and b). Fluorescence intensity line plots showed that the average length of EB1 and EB3 distribution along the microtubule lattice was 1.05±0.03 μm (mean±s.e.m., *n*=100) and 2.655±0.05 μm (mean±s.e.m., *n*=100), respectively (Figures 6b and c). Transfection of PC-3 or LNCaP C4-2B cells with either of two, independent, EB1- or EB3-specific siRNAs reduced EB1 and EB3 protein levels by greater than 85 and 70% respectively, as shown by quantitative immunoblotting (Figure 6d, Supplementary Figure S9A–F). EB1 knockdown did not change the length distribution of EB3 at the microtubule plus-end (Figures 6c and e). EB3 knockdown, however, increased the length of EB1 distribution along the microtubule to an average of 2.71±0.04 μm (mean±s.e.m., *n*=100; Figures 6c and e). EB1 or EB3 knockdown in PC-3 or LNCaP C4-2B cells reduced invasion of Matrigel in CXCL12 gradients, with EB3 knockdown having the greater effect (Figure 6f and Supplementary Figure S9G and H). Cell viability assays showed that the effect of EB-specific siRNA is not due to cell death (Supplementary Figure S5C). The effect of EB3 knockdown on prostate cancer cell invasion of Matrigel is consistent with the involvement of the drebrin/EB3 pathway in this process.

It has been reported that knockdown of EB1 produced no change in microtubule dynamics in undifferentiated myoblasts (C2C12 cells)^26^ or CHO-K1^27^ cells but shortened microtubules in Sertoli cells.^28^ EB3 knockdown produced curly microtubules in C2C12 cells^26^ but had no effect on dynamic microtubules in CHO-K1 cells.^27^ To test whether EB1 or EB3 knockdown affects microtubule dynamics in prostate cancer cell lines we assessed the morphology and distribution of dynamic microtubules in cells lacking EB1 or EB3 by labelling with an antibody against dynamic microtubules. This showed that neither EB1 nor EB3 knockdown affected the morphology of dynamic microtubules or prevented microtubules from extending to the cell periphery (Supplementary Figure S10) implying that the effects of EB3 knockdown on prostate cancer cell invasion are primarily due to loss of EB3 from microtubules and not secondarily due to changes in microtubule dynamics.

## Discussion

We show here that drebrin is expressed in basal, but not luminal, epithelial cells in normal human prostate glands and is upregulated in luminal epithelial cells in malignant glands. An upregulation of drebrin in malignant tissue was confirmed by quantitative immunoblotting. We also found high expression of drebrin in PC-3 and LNCaP C4-2B cells, which are human prostate cancer cell lines isolated from metastatic tissue, further supporting the idea that drebrin expression might be dysregulated in prostate cancer and contribute to metastatic activity. Drebrin is dysregulated in several cancers.^29, 30, 31, 32, 33^ Genomic analysis implicates drebrin copy number gain in 11% of prostate cancer cases.^19^ Our observation that drebrin mRNA upregulation and copy number gain occurs significantly more frequently in prostate cancer metastasis samples than in material from primary prostate cancers raises the possibility that an increase in drebrin expression could promote prostate cancer progression, though it is also possible that these genetic events may be a consequence of metastasis formation, perhaps reflecting changes in the tumour microenvironment. p35 and Cdk5 are also upregulated in prostate cancer and prostate cancer cell lines and Cdk5 activity has previously been shown to contribute to prostate cancer cell motility.^34^ These findings suggest, therefore, that the drebrin/EB3 pathway as a whole is dysregulated in prostate cancer.

We found evidence that the drebrin/EB3 pathway is important in prostate cancer cell invasion and the chemotactic response to the chemokine CXCL12. BTP2, an inhibitor of drebrin F-actin-binding, and drebrin and EB3 knockdown impaired prostate cancer cell invasion whereas drebrin and EB3 over-expression enhanced it. Drebrin binds to CXCR4 and recruits CXCR4 to the immunological synapse.^17^ CXCL12 signalling can promote prostate cancer cell invasion through an extracellular matrix, and trans-migration across bone marrow endothelial cell monolayers.^13^ Furthermore, blockade of CXCR4 prevents metastasis in prostate metastatic *in vivo* models.^15^ In line with our work and other studies of prostate cancer, the CXCL12/CXCR4 pathway has also been shown to increase the chemotactic and invasive behaviour of breast cancer cells and silencing CXCR4 prevents breast cancer metastatic spread in experimental mouse models.^35, 36, 37, 38^ Our experiments, however, do not answer the question of whether drebrin facilitates prostate cancer cell invasion through its role in CXCL12/CXCR4 signalling, or by coupling F-actin to dynamic microtubules, or by both mechanisms. There is evidence that CXCL12/CXCR4 signalling associated with lipid rafts is important in driving prostate cancer cell metastasis in bone^39^ but there is no evidence that drebrin associates with lipid rafts.^40^ Co-ordination of the microtubule and F-actin cytoskeletons mediated by the drebrin/EB3 pathway occurs in filopodia leading to their stabilization in response to extrinsic cues.^3^ There is mounting evidence that filopodia are important in the polarization and guided movement of cancer cells in 3D and in cancer metastasis and invasion,^41^ an idea supported by the findings reported here.

An unexpected finding was that EB1 and EB3 occupy different regions along the microtubule lattice; EB1 has a shorter and more distal location while EB3 occupies a longer stretch of the microtubule lattice and is more proximal. EB1 recognizes GTP-bound tubulin in the GTP-cap region at the plus-end of microtubules.^42^ The more proximal binding of EB3 is consistent with EB3 recognizing GDP-bound tubulin. The differential localization of these two +TIP proteins on the same microtubule has implications for the consequences of drebrin binding to EB3. Because EB1 is more distally located on the microtubule lattice than EB3, the growth of a microtubule cross-linked through the drebrin/EB3 pathway to actin filaments at the base of a filopodium would probably not be impeded. As the microtubule extends into the filopodium, iterative proximo-distal addition of drebrin/EB3-mediated cross-links would produce a zippering-up effect of the microtubule to F-actin in the filopodium. Consistent with this, the dynamic behaviour of drebrin, in which drebrin extends proximo-distally into the filopodium, predicted by a zippering-up effect, has been observed.^2^

## Materials and Methods

### Antibodies and reagents

Cdk5 (Abcam, Cambridge, UK; clone 2G2), cytokeratin 5 (Abcam, EP1601Y), cytokeratin 8 (BioLegend, London, UK; clone 1E8), drebrin (GeneTex, Isleworth, UK; GTX11068), drebrin (Abcam 176318 & 178408), drebrin (Progen, Heidelberg, Germany; clone Mx823), EB1 (BD Transduction Laboratories, Oxford, UK; clone 5), EB3 (Millipore, Consett, UK; AB6033), p35 (Abcam, ab66064), GAPDH (GeneTex, clone GT239), GFP (Abcam, 6556), laminin (Sigma-Aldrich, Poole, UK; clone LAM-89), p35/25 (Cell Signaling, Hitchin, UK; C64B10), p63 (Abcam, ab735 clone BC4A4), pERK1/2 (Thr202/Tyr204) antibody (Cell Signaling; #9101), pS142-drebrin (Millipore, clone 3C14), tyrosinated α-tubulin (SeroTec, Kidlington, UK; clone YL 1/2), vimentin (Dako, Glostrup, Denmark; clone V9). Horseradish peroxidase-conjugated secondary antibodies were from Dako. Alexa-conjugated secondary antibodies and phalloidin were from Life Technologies, Warrington, UK. BTP2 (YM-58483) was from Cambridge Bioscience (Cambridge, UK; CAY13246). Recombinant human CXCL12 was from PeproTech (London, UK; 300-28A). Matrigel was from Corning (New York, USA; 354234) and fibronectin from Millipore.

### Cell culture

Human prostate cell lines (DU-145, LNCaP, LNCap C4-2B, PC-3 and PNT2-C2) were maintained in RPMI medium supplemented with 10% fetal bovine serum (Sigma-Aldrich), 1% Glutamax and penicillin/streptomycin (50 μg/ml; Life Technologies). All cell lines were grown in a humidified incubator at 37 °C and 5% CO_2._ Cell lines were from ECACC and replaced every 4 months.

### DNA constructs and transfections

The K270M, K271M drebrin-GFP mutant, CFP-EB1M and CFP-EB3M have been described.^2, 22^ Cells were transfected using X-tremeGENE HP (Roche, Welwyn Garden City, UK) following manufacturer’s instructions. Drebrin knockdown was achieved using ON-TARGETplus human drebrin siRNA (Dharmacon, Lafayette, USA; LU-011841-00-0002) with the following target sequences: GGAUUAACCGAGAGCAGUU (drebrin siRNA 1), CCUCAAGCUUGCAGCAUCA (drebrin siRNA 2) or ON-TARGETplus human drebrin SMARTpool siRNA (Dharmacon; L-011841-00-0005) with the following target sequences: GGAGGAGGCAGCAGCUAUU, GGAUUAACCGAGAGCAGUU, CCUCAAGCUUGCAGCAUCA, GGAGUUUGCCCAAUCGGAA. EB1 and EB3 knockdown was achieved using ON-TARGETplus human EB1 and EB3 siRNA (Dharmacon; LU-006824-00-0002 and LU-013109-00-0002) with the following target sequences, EB1: UGACAAAGAUCGAACAGUU (EB1 siRNA 1), AGAUGAAGGCUUUGUGAUA (EB1 siRNA 2), and EB3: CAGCAAACUUCGUGACAUC (EB3 siRNA 1), UGAGACUGAUGCCCAAAUU (EB3 siRNA 2) or ON-TARGETplus human EB1 and EB3 SMARTpool siRNA (Dharmacon; L-006824-00-0005 and L-013109-00-0005) with the following target sequences, EB1: GGAAAGCUACGGAACAUUG, AAACGACCCUGUAUUGCAG, UGACAAAGAUCGAACAGUU, AGAUGAAGGCUUUGUGAUA, and EB3: CCUCAACUAUACCAAGAUA, CAGCAAACUUCGUGACAUC, GUAGAGAAAUUAGUGAAAG, UGAGACUGAUGCCCAAAUU. An ON-TARGETplus non-targeting control pool was also purchased from Dharmacon (D-001810-10-05). Cells were transfected with control, drebrin, EB1 and EB3-specific oligos using HiPerFect Transfection Reagent (Qiagen, Germantown, MD, USA) following manufacturer's instructions.

### 3D *in vitro* Transwell invasion assay

Matrigel (5 mg/ml), in serum-free RPMI, was pipetted into a Transwell insert (Corning 3422, 8 μm pore diameter) and allowed to set at 37 °C for 1 h. Suspensions of siRNA-transfected, CFP-transfected or DMSO-, or BTP2-treated cells were prepared at 2.5 × 10^4^ cells in 200 μl of serum-free RPMI were pipetted on top of the Matrigel layer in the Transwell insert and 500 μl of serum-free RPMI containing 100 ng/ml CXCL12 was added to the lower chamber. DMSO or BTP2 were re-added to the treated cell suspensions before plating in the insert. After 48 h, cells that had reached the lower surface of the insert filters were fixed with 3% formaldehyde and 0.2% glutaraldehyde in PBS pH 7.2 and then stained with 0.1% w/v cresyl violet. Stained filters were viewed under an Olympus BH2 microscope and images captured with a DP70 digital camera. In the case of invasion assays with CFP-transfected cells, the cells were fixed as described previously but the filters were immunolabelled with GFP antibody followed by Alexa-conjugated secondary antibody and imaged using an Olympus FluoView confocal microscope. Cells were counted manually using ImageJ software (NIH, Bethesda, MD, USA).

### MTT viability and Matrigel adhesion assays

MTT viability assay was performed as described.^43^ For adhesion assays, cells were pre-incubated with DMSO or BTP2 in suspension at 37 ^o^C for 1 h. Glass coverslips were coated with ice-cold Matrigel (5 mg/ml) in serum-free RPMI and incubated at 37 ^o^C for 1 h to allow the gel to set. DMSO or BTP2 were re-added to the cell suspensions before cells were plated at a density of 5 × 10^4^ cells per well of a 24-well plate. Cells were allowed to adhere for 1.5 h at 37 ^o^C. Cells were then fixed with 3% formaldehyde and 0.2% glutaraldehyde in PBS pH 7.2. Cells, labelled with Alexa-conjugated phalloidin, were imaged using an Olympus FluoView confocal microscope. Cell numbers were counted manually using ImageJ.

### Live cell imaging

Cells were embedded in Matrigel in μ-slide chemotaxis^3D^ chambers (Ibidi, Glasgow, UK; IB-80326) following the manufacturer’s instructions. For drug treatments, cell suspensions were incubated at 37 °C for 1 h in the presence of the drug. Subsequently, cell suspensions were mixed with ice-cold Matrigel (5 mg/ml). DMSO and BTP2 were re-added at this stage. The cell/gel mixture (6 μl) was immediately pipetted into the central observation area of the chemotaxis chamber. The chamber was then covered with the lid and incubated in a humidified 10 cm Petri dish in an incubator at 37 °C for 30 min until the Matrigel had set. Both reservoirs either side of the observation area were then filled with 65 μl serum-free RPMI. To establish a chemotactic gradient across the chamber, 15 μl of 200 ng/ml CXCL12 in serum-free RPMI was pipetted into the bottom filling port of the left reservoir before removing 15 μl from the top port. This step was repeated once more so that the resulting concentration of chemoattractant in the left reservoir was 0.5 times the injected concentration, that is, 100 ng/ml. Time-lapse phase contrast imaging was performed with an Olympus IX70 inverted microscope equipped with an environmental chamber (Solent) using phase contrast objectives. Cultures were maintained at 37 °C and gassed with CO_2_. Images were captured with a Hamamatsu 9100 EM-CCD camera and processed in ImageJ.

For cell motility assays, PC-3 cells were cultured in opti-MEM Medium supplemented with 10% fetal bovine serum at 37 ^°^C in 6-well plates coated with fibronectin (10 μg/ml). Phase contrast images (100) at 10 min intervals were captured in the presence of DMSO (vehicle control) or different concentrations of BTP2. Speed of motility was quantified using the Cell Tracker plug-in of ImageJ.

### 3D chemotaxis chamber immunofluorescence

Cells in chemotaxis chambers were fixed for 20 min with 3% formaldehyde and 0.2% glutaraldehyde in PBS pH 7.2 at 37 °C or with 3% formaldehyde, 0.2% glutaraldehyde and 0.2% Triton-X-100 in PHEM buffer pH 6.9. Cells were washed with PBS and then incubated in blocking buffer (BB; 5% (v/v) normal horse serum, 5% (v/v) normal goat serum, 50 mM L-lysine and 0.2% (v/v) Triton X-100 in PBS pH 7.2) for 2 h at room temperature (RT) followed by incubation with primary antibodies in BB for 1 h at RT and then overnight at 4 °C. Cells were washed with PBS before incubation with Alexa-conjugated secondary antibodies and phalloidin in BB for 4 h at RT. After washing with PBS, cells were imaged using an Olympus FluoView confocal microscope.

### Immunoblotting

Proteins were extracted from frozen human prostate tissue using RIPA buffer (20 mM Tris-HCl pH 7.4, 150 mM NaCl, 1 mM EGTA, 1 mM EDTA, 20 mM NaF, 1% (v/v) Triton X-100, 0.1% (w/v) SDS, 0.5% (v/v) Na deoxycholate) supplemented with protease and phosphatase inhibitors (Sigma-Aldrich). Cultured cells were washed in PBS and lysed in either hot 5x Lamelli sample buffer or RIPA buffer. For RIPA lysed samples, protein concentration was determined using BioRad (Hemel Hempstead, UK) Protein Assay reagent and between 10 μg to 50 μg loaded into each well of SDS-PAGE gels. Gels were transferred onto polyvinylidene difluoride membranes (Thermo, Paisley, Scotland, UK) and blocked overnight in 5% milk/Tris-buffered saline Triton X-100. Primary antibodies were incubated for 1 h at 37 ^°^C or overnight at 4 °C and secondary antibodies for 1 h at RT. Blots were developed with enhanced chemiluminescent kits (Millipore) and either, imaged and analysed using a Li-Cor Odyssey Fc, or exposed to clear blue X-ray films (Photon Imaging Systems, Swindon, UK). Films were developed in a Fuji processor, scanned using an Epsom V500 flat-bed scanner and analysed in ImageJ.

### CXCL12 stimulation of PC-3 cells

PC-3 cells were plated at 1 × 10^5^ cells per well of a 6-well plate and incubated overnight at 37 °C in complete medium. Prior to CXCL12 stimulation, the complete medium was replaced with serum-free medium and cells incubated for a further 3 h at 37 °C. One well was left in complete medium. Cells were then treated with CXCL12 (100 ng/ml) in serum-free medium for 2, 5, 10 or 20 min. Cells were then lysed on ice with RIPA buffer. Lysates were run on Western blots and probed with pS142-drebrin antibody and pERK1/2 antibody, as a control for induction of protein phosphorylation.

### Immunofluorescence of cultured cells

Cells were plated onto glass coverslips (13 mm) coated with 10 μg/ml fibronectin (Sigma-Aldrich) and, following attachment, were fixed for 10 min either with 3% formaldehyde and 0.2% glutaraldehyde in PBS pH 7.2 or 3% formaldehyde, 0.2% glutaraldehyde and 0.2% Triton X-100 in PHEM buffer pH 6.9 at 37 °C. For EB1 and EB3 immunolabelling, cells were serum starved for 3 h before re-addition of serum for 1 h, to induce cell spreading, followed by fixation with methanol for 15 min at –20 ^o^C and 4% formaldehyde in PBS for 10 min. Cultures were then incubated in BB at RT for 1 h followed by incubation in primary antibody in BB at RT for 1 h. Cultures were then washed with PBS and incubated in Alexa-conjugated secondary antibodies and phalloidin, to visualize F-actin, in BB for 1 h at RT. Finally, cultures were washed with PBS and mounted in FluorSave anti-fade reagent (Millipore). Labelled cultures were imaged using an Olympus FluoView confocal microscope. Fluorescence intensity line plots of microtubules and EB proteins were acquired using the Line tool and analysed using Plot Profile in ImageJ.

### Immunofluorescence of human prostate tissue

Tissue was obtained from consenting patients following local ethical committee approval. Frozen sections of human prostate were fixed with 4% formaldehyde in PBS pH 7.2 at 4 ^o^C for 20 min. Sections were washed with PBS and then incubated in BB at RT for 1 h followed by incubation in primary antibody in BB at RT for 2 h. Sections were then washed with PBS and incubated in Alexa-conjugated secondary antibodies and phalloidin in BB at RT for 2 h. Labelled sections were viewed using an Olympus FluoView confocal microscope. Fluorescent images in TIFF format were manipulated using Adobe Photoshop.

### Immunohistochemistry of prostate tissue

Tissue microarray sections were obtained from the Oxford Centre for Histopathology Research, Oxford University Hospitals NHS Foundation Trust, Oxford, UK. Microscope slide-mounted tissue sections were deparaffinized, hydrated and exposed to a rabbit polyclonal antibody against drebrin (GeneTex, 1:500) or a mouse monoclonal antibody against pS142-drebrin^4^ (1:100) overnight following antigen retrieval using citrate buffer (10 mM sodium citrate, 0.05% Tween 20, pH 6.5) for 20 min at 90 °C in a microwave oven. Pre-diluted ImmPACT secondary antibody (Vector Labs, Peterborough, UK) was applied to the slides for 30 min and ImmPACT DAB (Vector Labs) was added to observe positive stain. Negative controls for immunohistochemistry included replacing primary antibody with horse serum. Samples were evaluated microscopically and digital photographs were taken with a 20 objective. Following immunohistochemistry, the maximum protein expression intensity in prostate epithelial cells (benign or malignant; 0–3, with 3=strong expression, 2=moderate expression, 1=weak expression and 0=no expression), and the percentage of prostate epithelial cells with that maximum expression (0–100%), was assigned for each core by a single expert uropathologist blinded to the study hypothesis. The maximum protein intensity (0–3) and percentage (0–100) scores were multiplied to give a final expression score for each core sample (range 0–300). The tissue microarray was built and this part of the study conducted under the Oxford Radcliffe Biobank ethics committee approval (reference number 09/H0606/5+5).

### Statistical analysis

*In vitro* data were analysed using GraphPad Prism 5 (GraphPad Software, La Jolla, CA, USA) or, for polar plots, Oriana 4 (Kovach Computing Services, Pentraeth, Wales, UK), and expressed as mean±s.e.m. The unpaired two-tailed Student’s *t-*test or ANOVA test was used for statistical analysis. Differences between values in all statistical tests were considered significant if *P*<0.05. Associations between mRNA expression or copy number groups and sample type (primary or metastasis) from the MSKCC Prostate Cancer Genomics Data Portal were investigated using the *χ*^2^-test.
