# Supplementary figures and images for: The drebrin/EB3 pathway drives invasive activity in prostate cancer
Source: Oncogene. 2017 Mar 20;36(29):4111–23. doi: 10.1038/onc.2017.45 (PMC5537610; doi:10.1038/onc.2017.45)

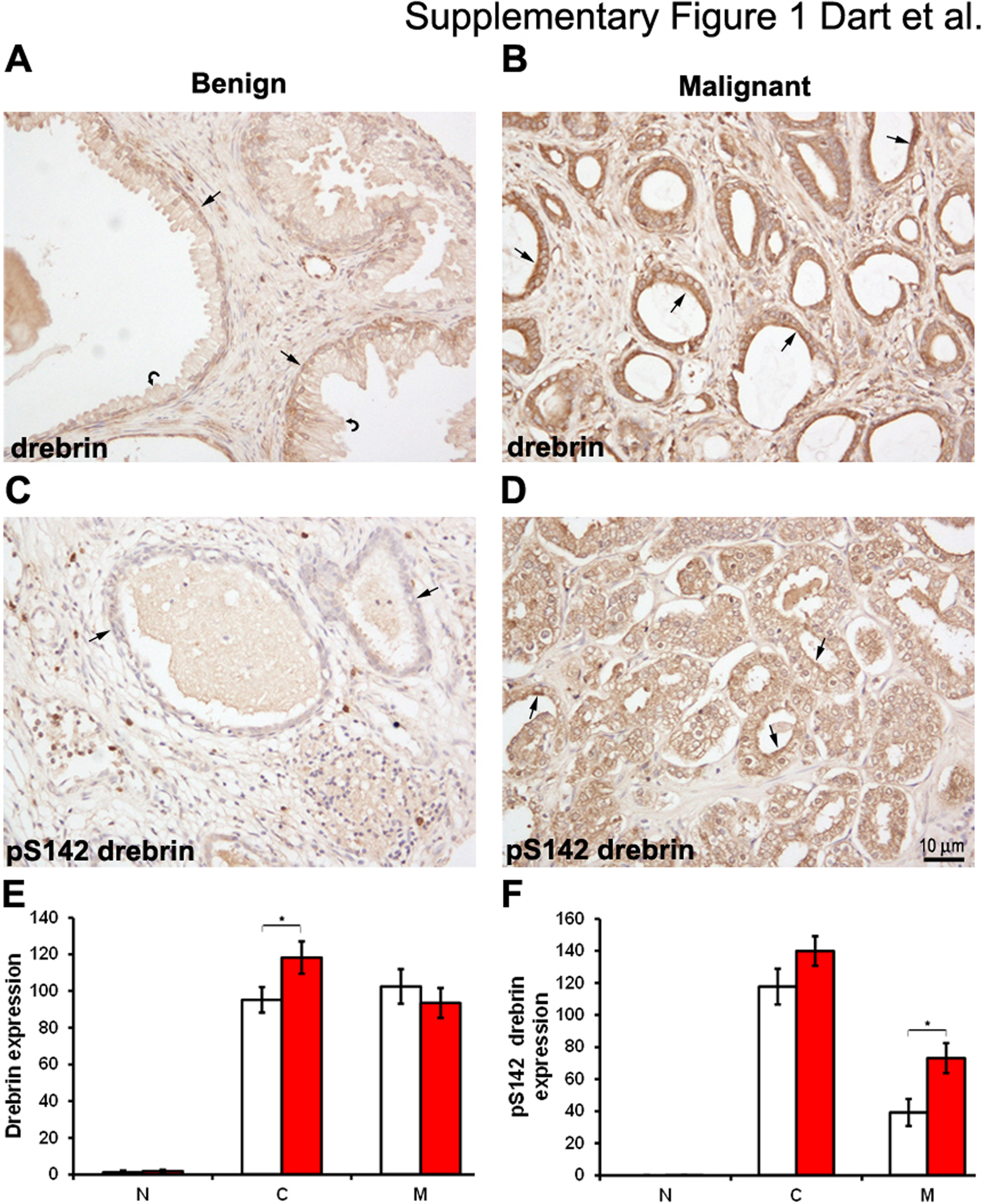

Supplement: Supplementary Figure S1 [file onc201745x1.tif]

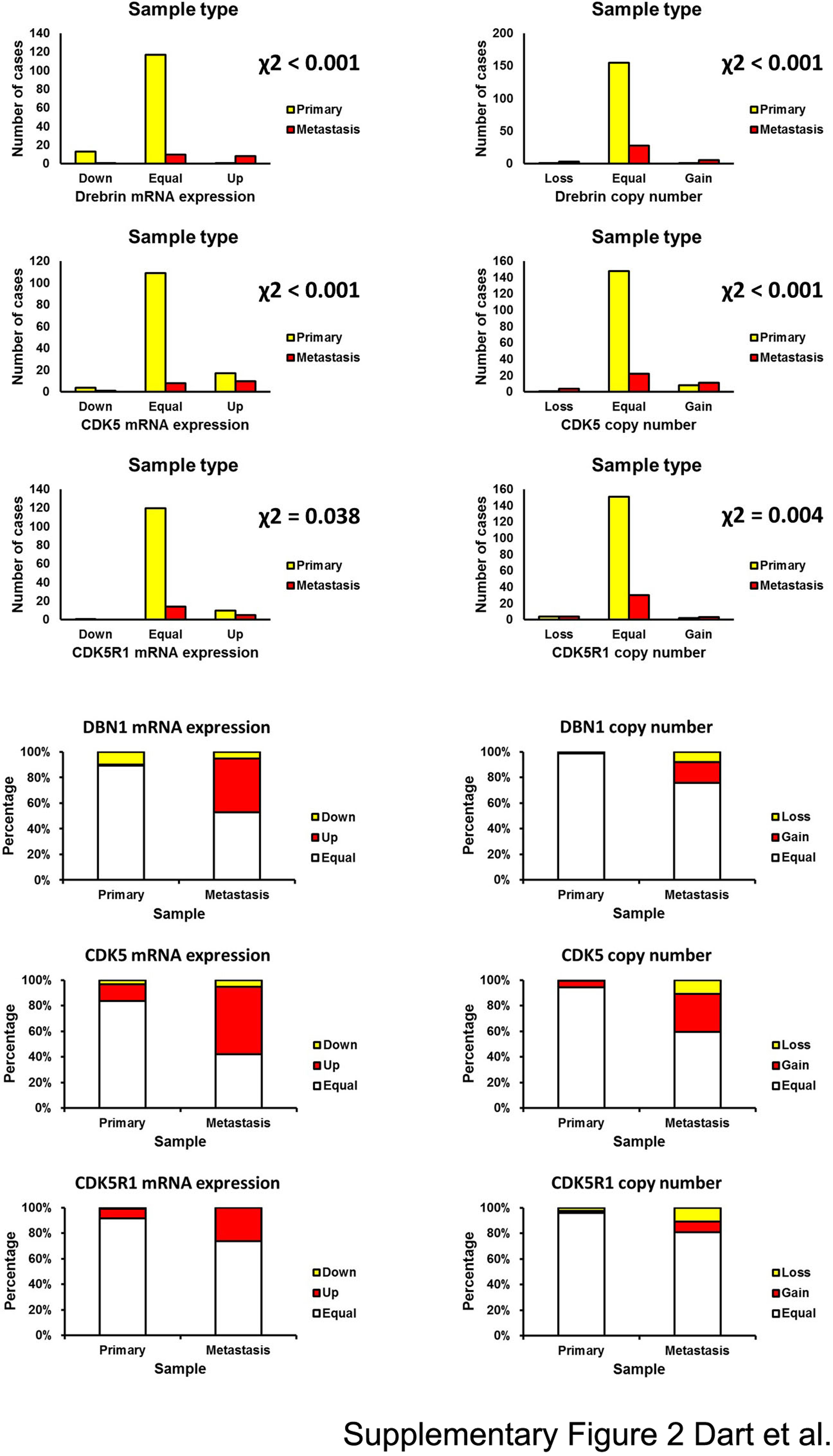

Supplement: Supplementary Figure S2 [file onc201745x2.tif]

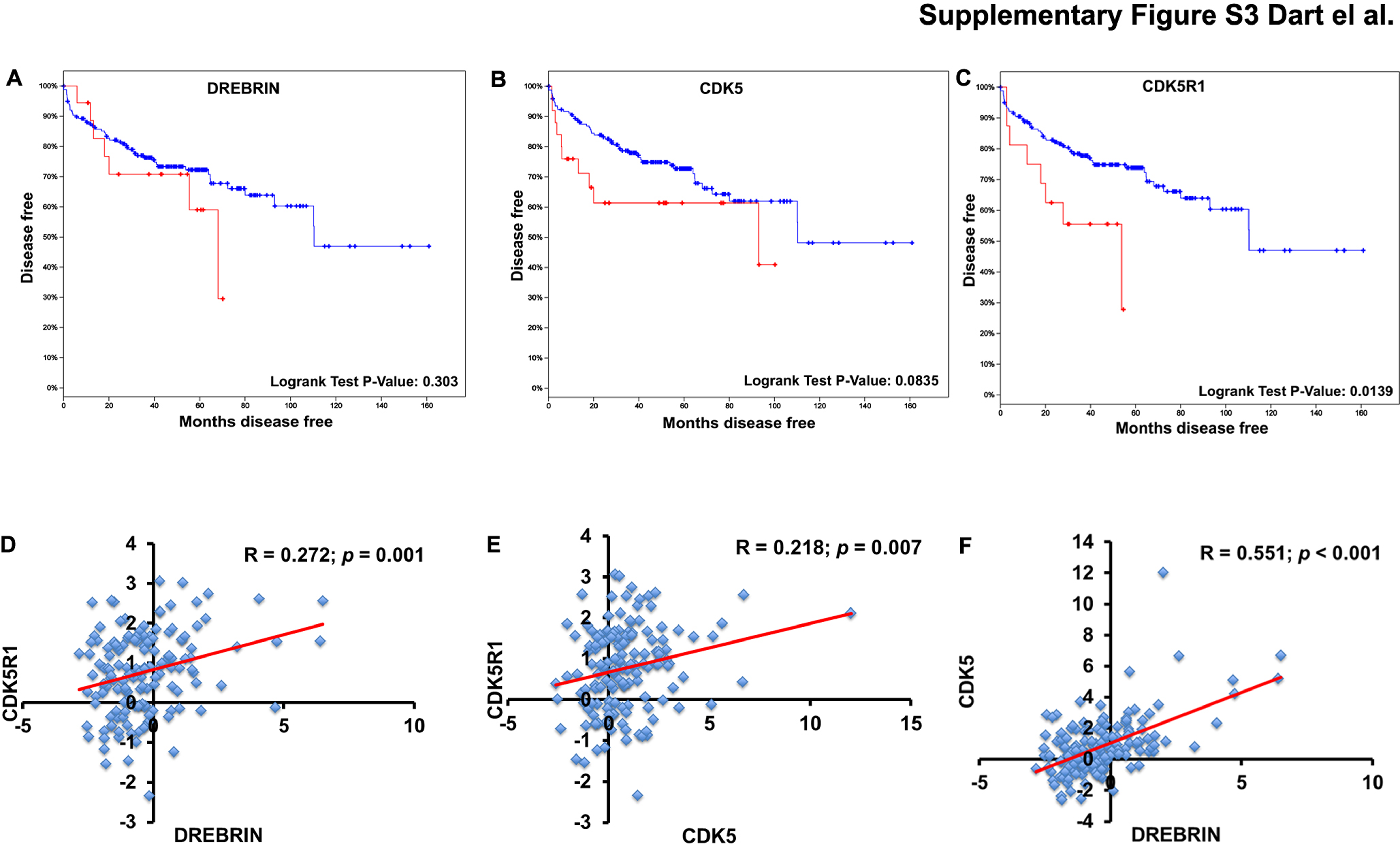

Supplement: Supplementary Figure S3 [file onc201745x3.tif]

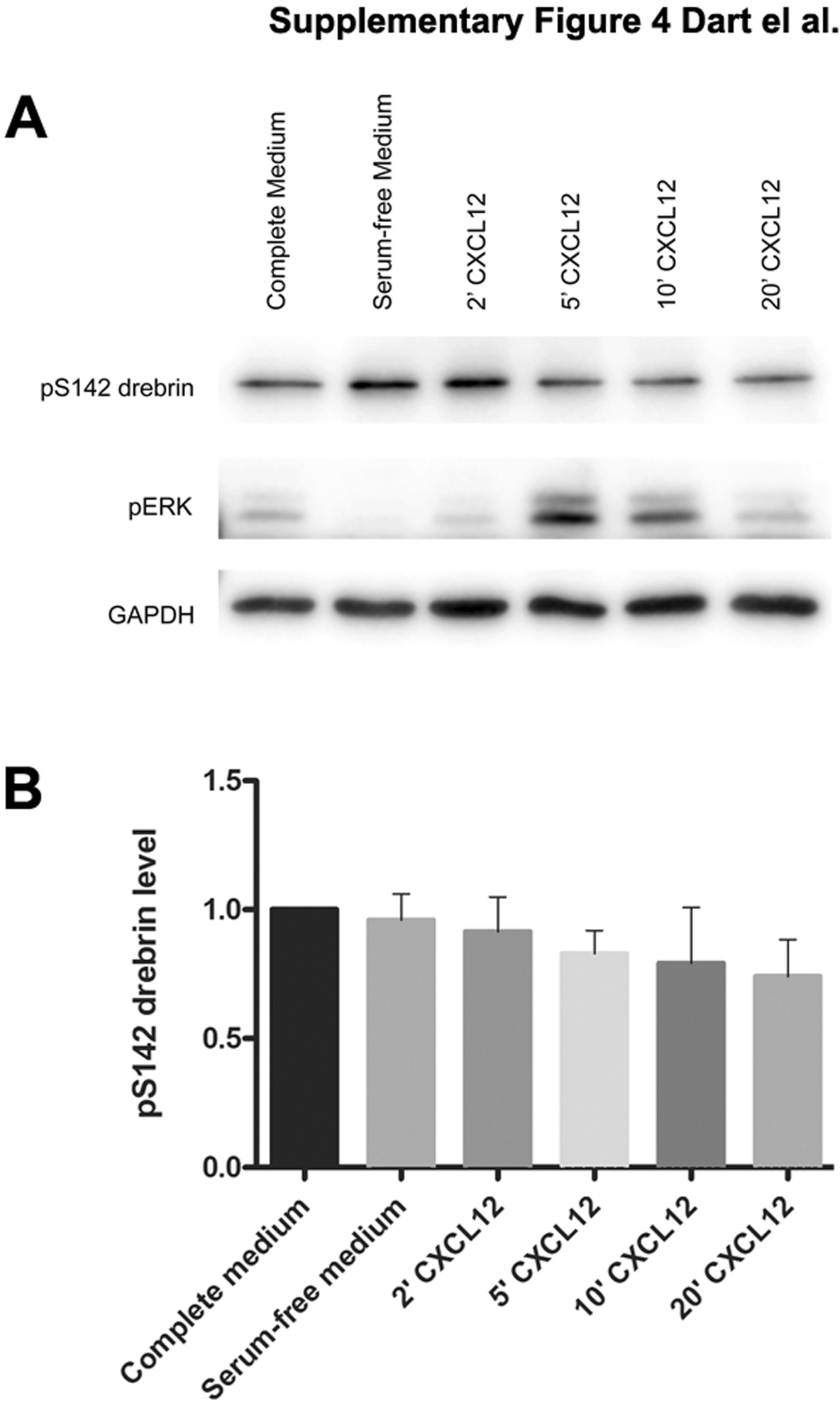

Supplement: Supplementary Figure S4 [file onc201745x4.tif]

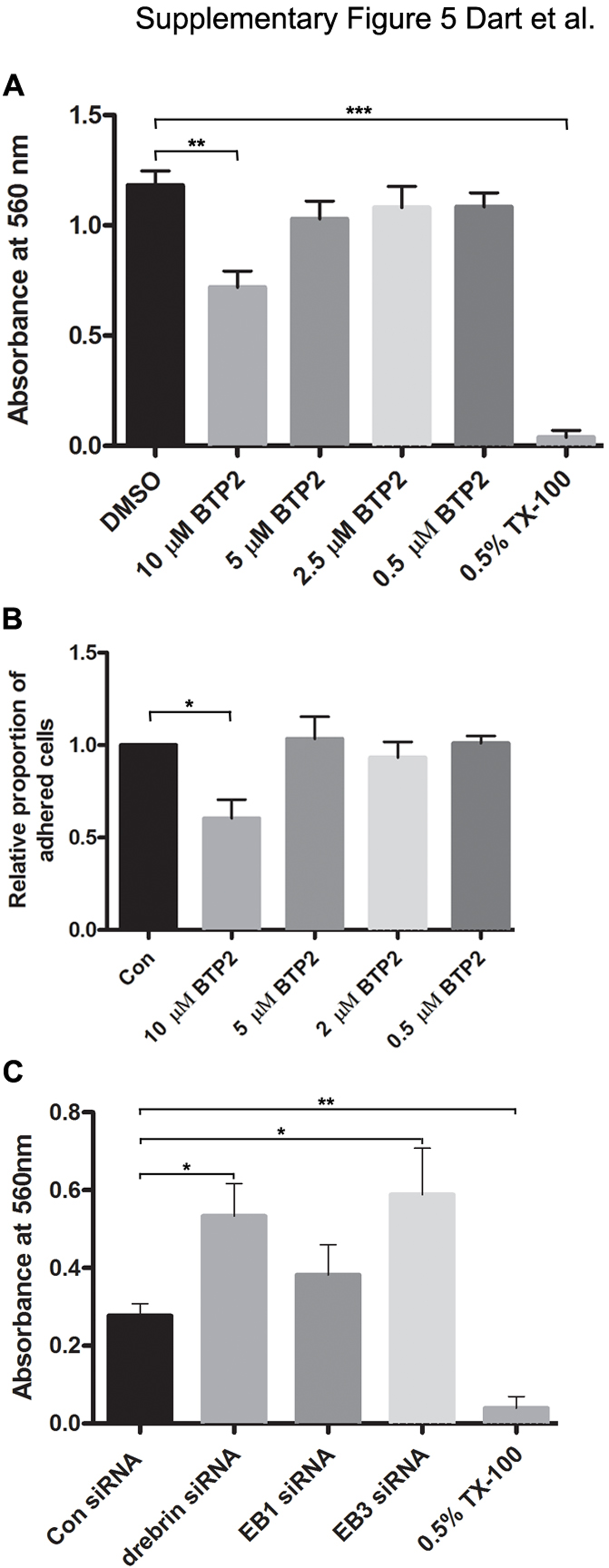

Supplement: Supplementary Figure S5 [file onc201745x5.tif]

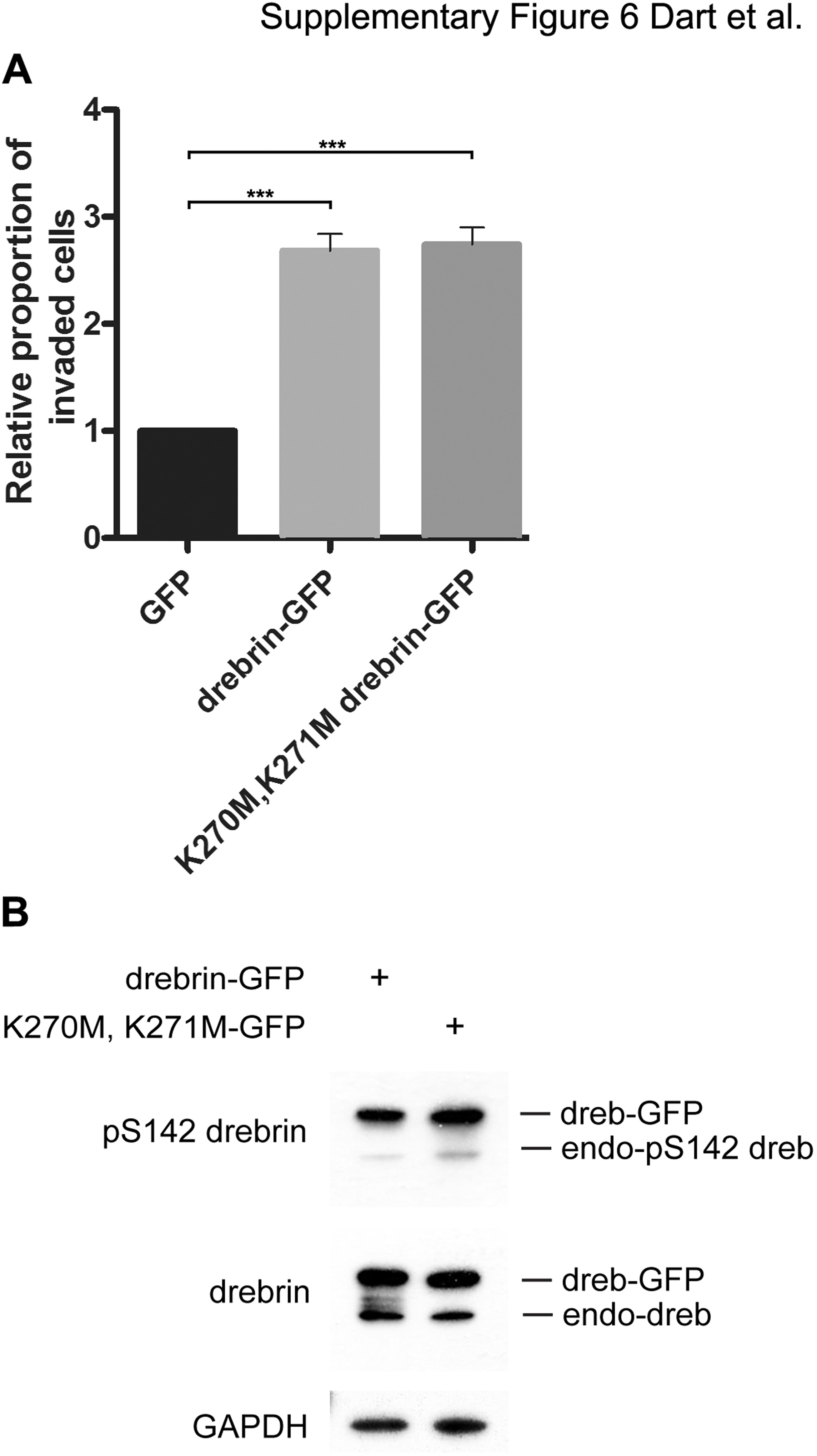

Supplement: Supplementary Figure S6 [file onc201745x6.tif]

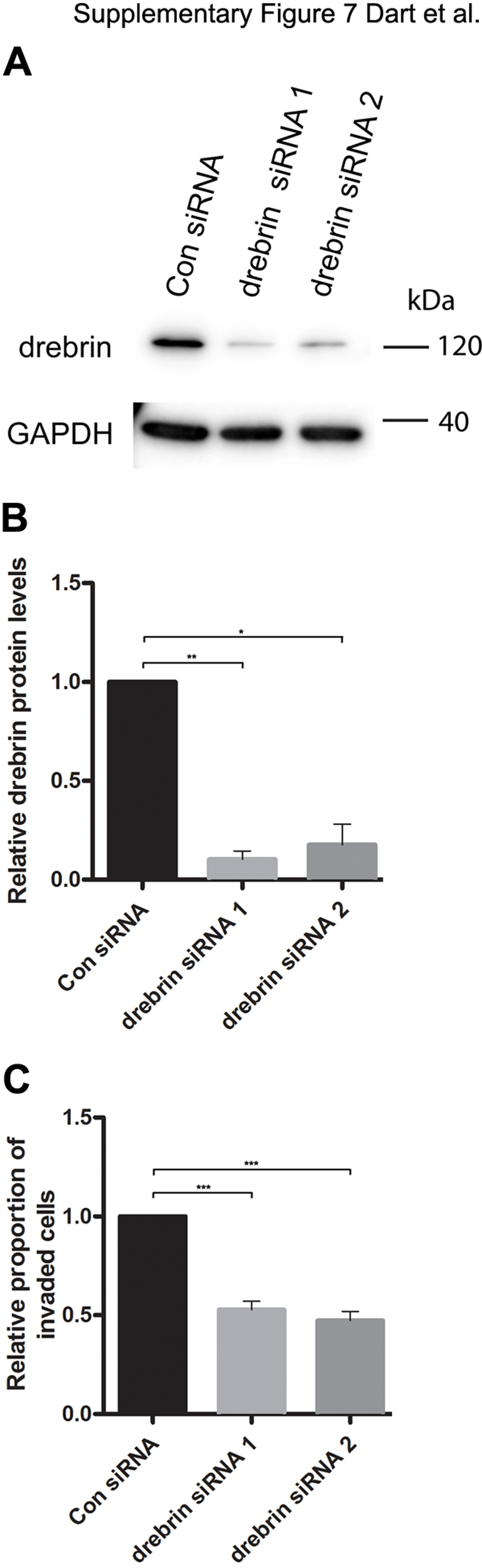

Supplement: Supplementary Figure S7 [file onc201745x7.tif]

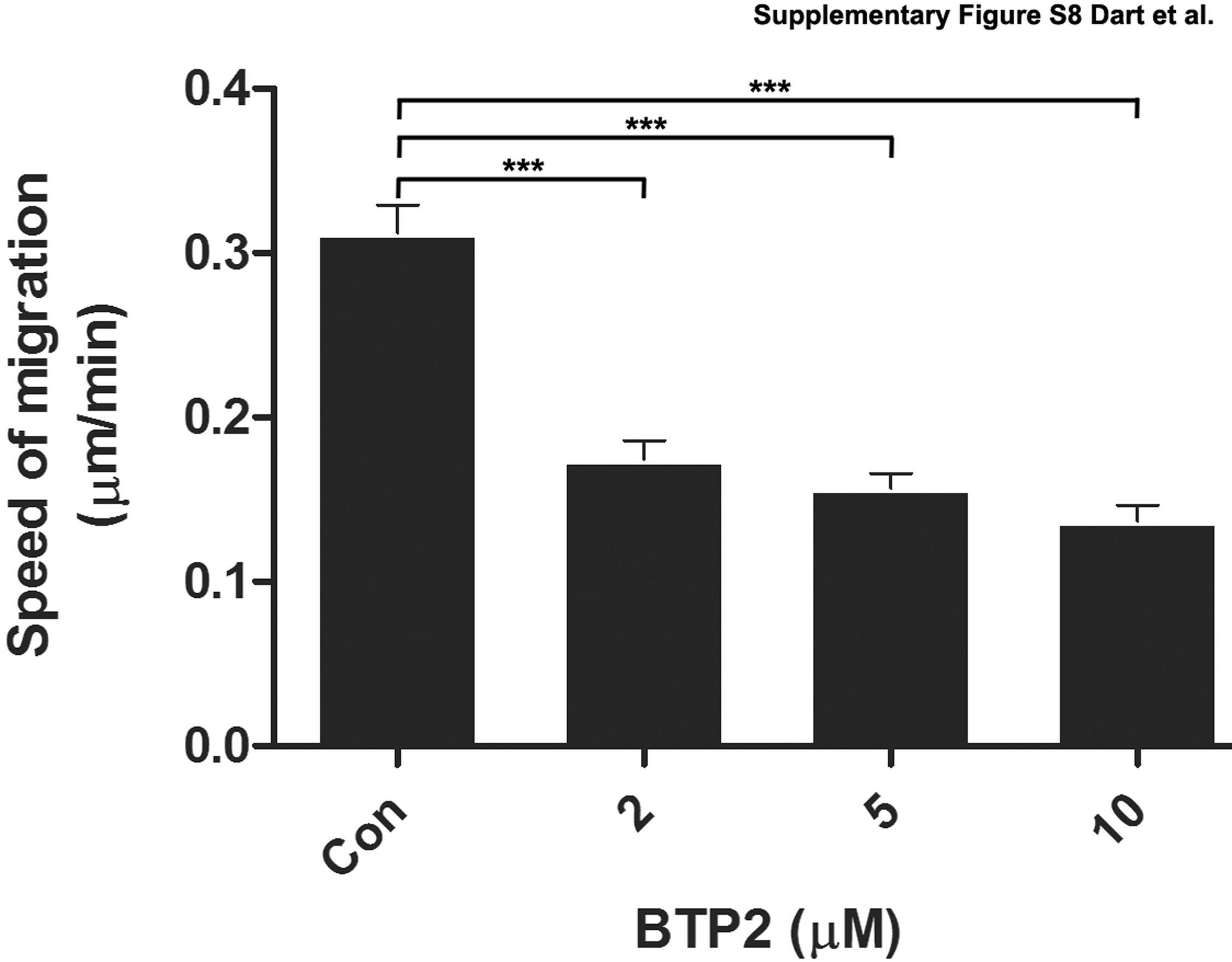

Supplement: Supplementary Figure S8 [file onc201745x8.tif]

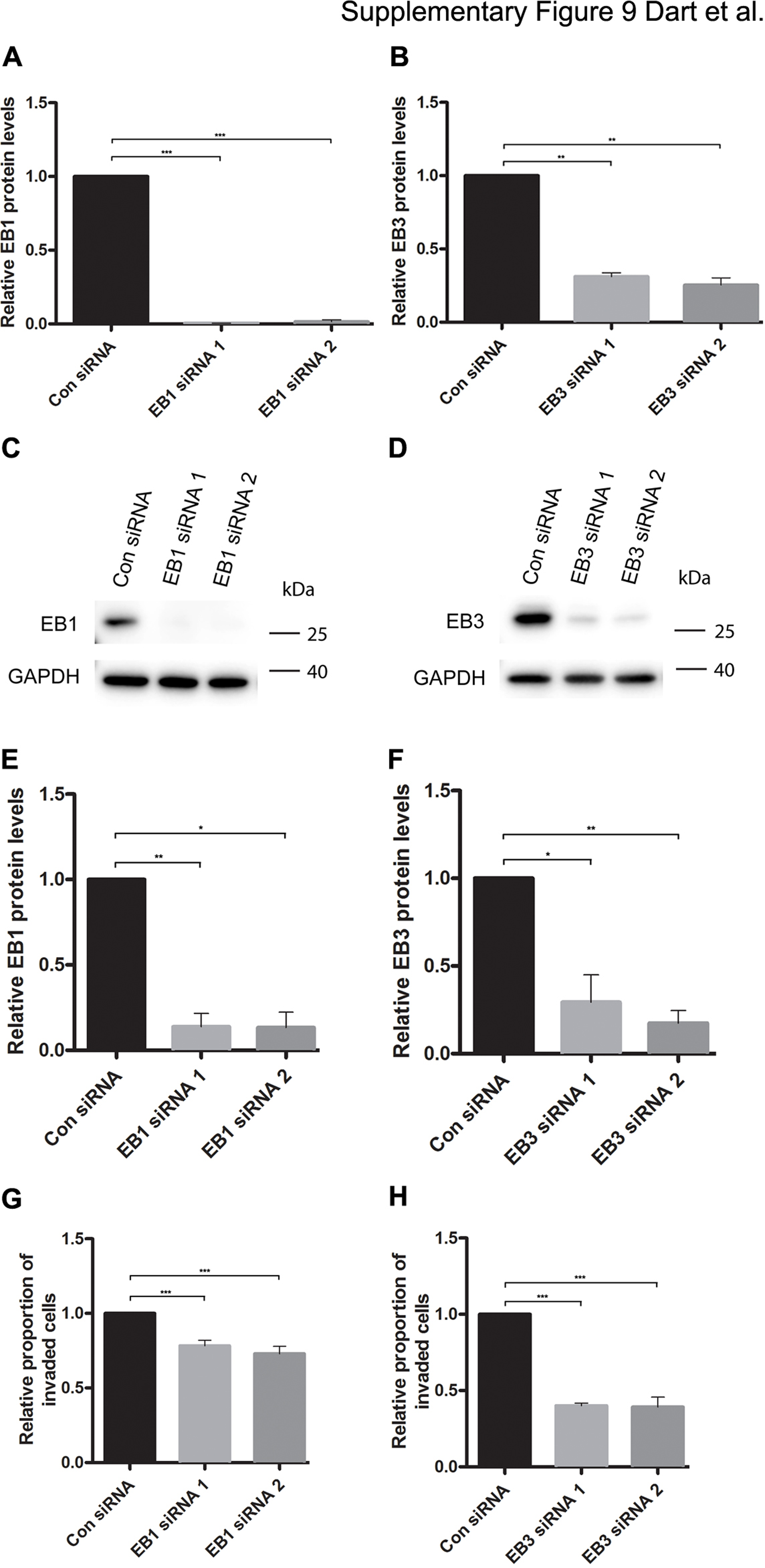

Supplement: Supplementary Figure S9 [file onc201745x9.tif]

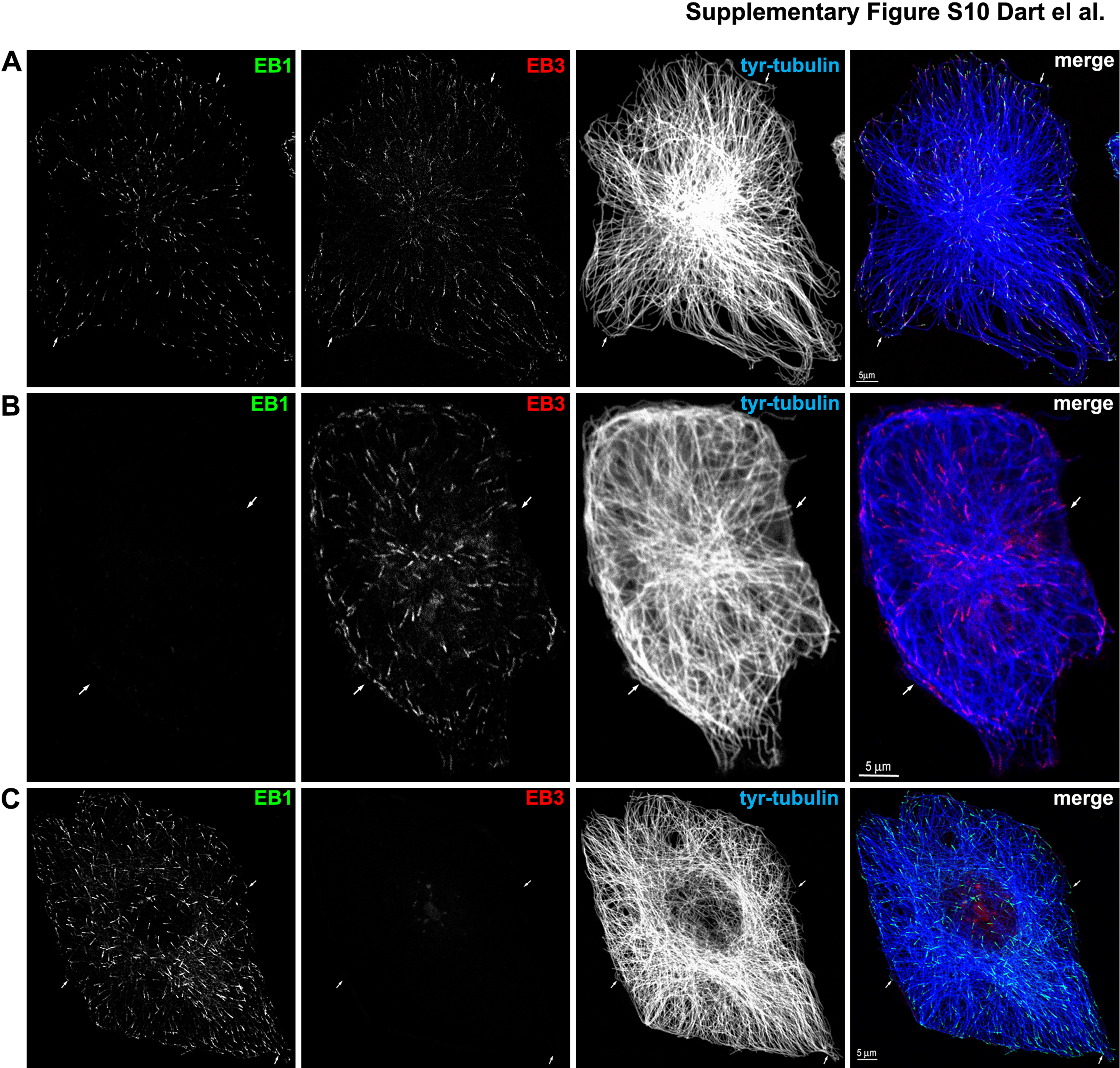

Supplement: Supplementary Figure S10 [file onc201745x10.tif]
