# Supplementary material for: The drebrin/EB3 pathway drives invasive activity in prostate cancer
Source: Oncogene. 2017 Mar 20;36(29):4111–23. doi: 10.1038/onc.2017.45 (PMC5537610; doi:10.1038/onc.2017.45)
Supplement: Supplementary Figure Legends [file onc201745x11.doc]

**Supplementary Figure Legends**

**Supplementary Figure S1.**

Cytoplasmic drebrin and plasma membrane pS142-drebrin are over-expressed in malignant prostate compared with benign prostate. A-D, Micrographs of the TMA showing benign (A, C) and malignant (B, D) human prostate tissue immunolabelled with antibodies to drebrin (A, B) or pS142-drebrin (C, D). A, In benign prostate glands, drebrin is expressed in basal epithelial cells (arrows). Luminal epithelial cells in these benign glands are not labelled with drebrin antibody (curved arrows). In contrast, in malignant tissue (B), drebrin is expressed throughout the epithelium (arrows). C, In benign prostate glands, pS142-drebrin is weakly expressed in basal epithelial cells (arrows) whereas in malignant tissue (d) pS142-drebrin is expressed in luminal epithelial cells, particularly at the plasma membrane (arrows). E, F, Semi-quantitative immunohistochemical analysis of drebrin (E) and pS142-drebrin (F) expression in the nucleus (N), cytoplasm (C) and plasma membrane (M) of benign prostate epithelial cells (white bars) and prostate cancer cells (red bars) from the TMA. Cytoplasmic drebrin and plasma membrane pS142-drebrin were significantly over-expressed in prostate cancer cells compared with benign prostate epithelial cells. Error bars are mean ± SEM from samples taken from at least 40 men with benign prostate hyperplasia and 110 men with prostate cancer. Significant difference: *, P<0.01.

**Supplementary Figure S2.**

Histograms showing the levels of drebrin, Cdk5 and Cdk5R1 (p35) mRNA expression and copy number in prostate cancer from the publically available MSKCC Prostate Cancer Genomics Data Portal dataset^19^. Increased mRNA expression and copy number gain of drebrin, Cdk5 and Cdk5R1 occur significantly more frequently in prostate cancer metastases than in primary prostate cancer cases (X^2^ < 0.05 for each).

**Supplementary Figure S3.**

Analysis of genetic alteration (A, drebrin; B, Cdk5; and C, Cdk5R1) and prostate cancer disease-free survival from the publically available MSKCC Prostate Cancer Genomics Data Portal dataset^19^ reveals that a genetic alteration in Cdk5R1 expression is associated with significantly reduced prostate cancer disease-free survival (*p* = 0.0139). A similar trend is observed for genetic alterations in drebrin and Cdk5, although the disease-free survival Kaplan-Meier estimate does not reach statistical significance for these latter two genes. Data are from patients with (red line) and without (blue line) overexpression/genetic alteration. A Pearson correlation analysis of the MSKCC dataset reveals that significant positive correlations can be observed between mRNA expression levels of drebrin, Cdk5 and Cdk5R1 (D, E, F) in prostate cancer samples.

**Supplementary Figure S4.**

A, Immunoblots of protein lysates from PC-3 cells treated with CXCL12 (100 ng/ml) for 2, 5, 10 or 20 minutes following a medium change to serum-free medium. The blots were probed with antibodies to pS142 drebrin, pERK and GAPDH, as a loading control. B, Quantification of relative pS142 drebrin protein levels from immunoblots of PC-3 cells treated with CXCL12. Error bars are mean ± SEM from 3 independent experiments.

**Supplementary Figure S5.**

A, Histogram showing the effects of BTP2 on PC-3 cell viability using the MTT assay. Error bars are mean ± SEM from 3 independent experiments each of two replicates per condition. Significant differences: **, P<0.01; ***, P<0.001. B, Histogram showing the relative proportion of PC-3 cells adhering to Matrigel following treatment with DMSO (Con) or BTP2. Error bars are mean ± SEM from 3 independent experiments. Significant difference: *, P<0.05. C, Histogram showing the effects of drebrin, EB1 and EB3 knockdown with siRNA on PC-3 cell viability using the MTT viability assay. Error bars are mean ± SEM from 3 independent experiments each of two replicates per condition. Significant differences: *, P<0.05; **, P<0.01.

**Supplementary Figure S6.**

A, Effects of expression of GFP, drebrin-GFP or K270M, K271M drebrin-GFP on PC-3 cell invasion in a 3D invasion assay in the presence of CXCL12 chemotactic gradients. Cells were transfected with plasmids before seeding onto Matrigel in the Transwell insert. After 48 hours, cells on the lower surface of the insert membrane were stained with cresyl violet and counted. Error bars are mean ± SEM from 3 independent experiments each of two replicates per condition. Significant difference: ***, P<0.001. B, The K270M, K271M drebrin-GFP mutant becomes phosphorylated at S142 when expressed in PC-3 cells. Immunoblots of PC-3 cells transfected with wild type drebrin-GFP or K270M, K271M drebrin-GFP and probed with antibodies against pS142-drebrin, drebrin and GAPDH. The drebrin antibodies recognise endogenous drebrin (endo-dreb) and the expressed protein (dreb-GFP).

**Supplementary Figure S7.**

A, Immunoblots of LNCaP C4-2B cells transfected with either control siRNA (Con siRNA) or drebrin siRNA 1 or drebrin siRNA 2 and probed with antibodies against drebrin and GAPDH, as a loading control. B, Quantification of relative drebrin protein levels from immunoblots of LNCaP C4-2B cells transfected with control siRNA (Con siRNA) or drebrin siRNA 1 or drebrin siRNA 2. Drebrin-specific siRNAs knock down drebrin levels by >85% compared to control. Error bars are mean ± SEM from 2 independent experiments. Significant differences: *, P<0.05, **, P<0.01. C, Effects of siRNA knockdown of drebrin on LNCaP C4-2B cell invasion in a 3D invasion assay in the presence of CXCL12 chemotactic gradients. Cells were transfected with control siRNA (Con siRNA) or drebrin siRNA 1 or drebrin siRNA 2 before seeding onto Matrigel in the Transwell insert. After 48 hours, cells on the lower surface of the insert membrane were stained with cresyl violet and counted. Error bars are mean ± SEM from 3 independent experiments each of two replicates per condition. Significant difference: ***, P<0.001.

**Supplementary Figure S8.**

Histogram showing the effects of BTP2 on PC-3 cell random motility in 2D. PC-3 cells were cultured in 6-well plates in the presence of DMSO (Con) or BTP2 at 2, 5 or 10 µM. Error bars are mean ± SEM from 3 or more replicates and a total of 130 or more cells. Significant differences: ***, P<0.001. 2, 5 and 10 µM BTP2 are not significantly different from each other.

**Supplementary Figure S9.**

A, Quantification of relative protein levels for EB1 from immunoblots of PC-3 cells transfected with control siRNA (Con siRNA), EB1 siRNA 1 or EB1 siRNA 2. In EB1 siRNA-transfected cells, EB1 levels are reduced to 4.5% of controls. Error bars are mean ± SEM from 3 independent experiments. Significant difference: ***, P<0.001. B, Quantification of relative protein levels for EB3 from immunoblots of PC-3 cells transfected with control siRNA (Con siRNA), EB3 siRNA 1 or EB3 siRNA 2. In EB3 siRNA-transfected cells, EB3 levels are reduced to 35% of controls. Error bars are mean ± SEM from 3 independent experiments. Significant difference: **, P<0.01. C, Immunoblots of LNCaP C4-2B cells transfected with either control siRNA (Con siRNA) or EB1 siRNA 1 or EB1 siRNA 2 and probed with antibodies against EB1 and GAPDH, as a loading control. D, Immunoblots of LNCaP C4-2B cells transfected with either control siRNA (Con siRNA) or EB3 siRNA 1 or EB3 siRNA 2 and probed with antibodies against EB3 and GAPDH, as a loading control. E, Quantification of relative protein levels for EB1 from immunoblots of LNCaP C4-2B cells transfected with control siRNA (Con siRNA), EB1 siRNA 1 or EB1 siRNA 2. In EB1 siRNA-transfected cells, EB1 levels are reduced to 10% of controls. Error bars are mean ± SEM from 3 independent experiments. Significant differences: *, P<0.05, **, P<0.01. F, Quantification of relative protein levels for EB3 from immunoblots of LNCaP C4-2B cells transfected with control siRNA (Con siRNA), EB3 siRNA 1 or EB3 siRNA 2. In EB3 siRNA-transfected cells, EB3 levels are reduced to 35% of controls. Error bars are mean ± SEM from 3 independent experiments. Significant differences: *, P<0.05, **, P<0.01. G, Effects of EB1 knockdown with siRNA on LNCaP C4-2B cell invasion in a 3D invasion assay in the presence of CXCL12 chemotactic gradients. Cells were transfected with control siRNA (Con siRNA), EB1 siRNA 1 or EB1 siRNA 2 before seeding onto Matrigel in the Transwell insert. After 48 hours, cells on the lower surface of the insert membrane were stained with cresyl violet and counted. Error bars are mean ± SEM from 3 independent experiments each of two replicates per condition. Significant difference: ***, P<0.001. H, Effects of EB3 knockdown with siRNA on LNCaP C4-2B cell invasion in a 3D invasion assay in the presence of CXCL12 chemotactic gradients. Cells were transfected with control siRNA (Con siRNA), EB3 siRNA 1 or EB3 siRNA 2 before seeding onto Matrigel in the Transwell insert. After 48 hours, cells on the lower surface of the insert membrane were stained with cresyl violet and counted. Error bars are mean ± SEM from 3 independent experiments each of two replicates per condition. Significant differences: ***, P<0.001.

**Supplementary Figure S10.**

Knockdown of EB1 or EB3 in PC-3 cells does not change the morphology of dynamic microtubules or prevent them from reaching the cell periphery. Immunofluorescence images of PC-3 cells from cultures transfected with control siRNA (A), EB1 siRNA (B) or EB3 siRNA (C) and labelled with antibodies against EB1, EB3 and tyrosinated α-tubulin (tyr-tubulin) to identify dynamic microtubules. In control cells (A) and in cells lacking either EB1 (B) or EB3 (C), dynamic microtubules have a normal morphology and extend as far as the cell periphery.
